# Taking Charge Against the Silent Pandemic—Electrochemical Characterization of Drug Resistance

**DOI:** 10.3390/molecules31193566

**Published:** 2026-10-07

**Authors:** Fangqing Liu, Michael A. Saley, Ana Carolina Mendes Hacke, Shubhneet Thind, Pauline Anne Barraquiel, Sabine Kuss

**Affiliations:** Department of Chemistry, University of Manitoba, Winnipeg, MB R3T 2N2, Canadamichael.saley@umanitoba.ca (M.A.S.); barraqpa@myumanitoba.ca (P.A.B.)

**Keywords:** electrochemical methods, biosensors, antimicrobial resistance, chemoresistance, point-of-care devices

## Abstract

The occurrence of antimicrobial resistance (AMR) and chemoresistance (CMR) during bacterial infections and cancer progression, respectively, has become a serious threat leading to increased mortality rates and putting significant pressure on healthcare systems globally. The design and application of new bioanalytical methodologies capable of early AMR and CMR detection in patients is urgently needed to overcome the challenges imposed by drug resistance. Electrochemical methods and (bio)sensors have attracted the widespread attention of researchers as promising tools to identify AMR and CMR in patients due to their high sensitivity, operational simplicity, lower cost, and fast response when compared to conventional assays. In addition, these analytical tools can be miniaturized, favoring the design of point-of-care (POC) devices. In this feature review paper, we report the most recent and promising achievements of electrochemistry-based approaches to detect and understand AMR and CMR, and answer the question of whether genomic or activity-based methods are more advantageous for drug susceptibility testing. The use of methods such as voltammetry, electrochemical impedance spectroscopy, and scanning electrochemical microscopy (SECM) is highlighted and discussed. In addition, technical challenges related to the widespread commercialization of such electrochemical devices are discussed, and future research directions are suggested.

## 1. Introduction

Bacterial infections are among the main causes of death worldwide. It is estimated that around 7.7 million people die every year due to diverse bacterial infections, which make them the second-leading cause of death globally [1]. The World Health Organization (WHO) states that there is an urgent need for the development of strategies to manage bacterial infections [2], increasing treatment efficiency and patient survival. Antibiotics are undeniably the most effective way to treat bacterial infections, which allows this class of pharmaceuticals to save millions of lives every year [3]. However, bacteria naturally develop molecular strategies to overcome the action of antibiotics and survive in their presence [4], a process that can be accelerated with the over-usage of antibiotics by the health care system, contamination of groundwater with antibiotics [5], or exposure to antibiotics through agricultural products such as milk and eggs [6]. This concerning issue has been significantly growing around the globe over the years, and recent statistics show that nearly 5 million deaths were caused by infections derived from resistant bacteria in 2019. Alarming projections show that antimicrobial resistance (AMR) could cause 10 million deaths per year by 2050 [7], which makes it one of the main public health threats of the present century [8].

In a similar way to bacteria, cancer cells also become resistant to the action of chemotherapeutics, which quickly leads to treatment inefficacy and dramatic increases in mortality rates. It is estimated that, among the 10 million deaths caused by cancer worldwide in 2020 [9], 90% were directly or indirectly related to drug-resistant tumors [10]. This feature is known as chemoresistance (CMR), and it has been reported for almost all drugs utilized to treat deadly types of cancer, including breast, gastric, colorectal and lung cancer [11]. Tumors can develop drug resistance after chemotherapy is initiated (acquired resistance) or even before any therapeutic intervention is attempted (intrinsic resistance) [12]. Therefore, CMR in cancer patients has become one of the main issues that need to be understood and overcome by oncology research to increase chemotherapy effectiveness and improve patient outcomes [13].

Besides increasing mortality rates, drug resistance in cancer and bacterial infections exerts significant pressure on healthcare systems and causes enormous economic losses globally. Patients affected by a resistant disease usually face longer hospital stays, require higher costs for medication, and present a much slower recovery rate, which ultimately leads to a pronounced loss in healthcare efficiency. It is estimated that these two conditions have contributed to a loss of almost US$47 trillion globally from 2010 to 2023 [14]. To prevent these negative impacts from further escalation, strategies to mitigate the health and economic impacts of AMR and CMR need to be urgently advanced. The key to addressing such issues consists mainly in understanding the molecular mechanisms that culminate in drug resistance and developing strategies to overcome them [3,11]. Drug resistance can arise through several molecular mechanisms, depending on the type of drug and the organism involved. These mechanisms can occur individually or in combination, leading to varying degrees of resistance to different drugs. The capacity to identify and elucidate the details of these mechanisms is crucial for developing strategies to combat drug resistance effectively [11,12,15]. The most common cellular defense mechanisms are drug efflux pumps, which actively remove drugs from the cell interior, reducing the concentration of the drug inside the cell and thereby decreasing its effectiveness; cell membrane modifications, which prevent drugs from entering an organism; target mutation, where pathogens can develop resistance by altering the genes encoding for drug targets (e.g., enzymes, receptors) to alter the target site so that the drug no longer binds effectively; and chemical modification of the drug target molecules. Additionally, bacteria can form biofilms, which are resistant to antibiotics due to physical barriers and altered metabolic states of cells. The early detection of AMR and CMR, the use of combination therapy, and the addition of new compounds in treatment strategies, such as natural products, are some of the most promising strategies to overcome drug resistance in patients [3,11].

But how does one determine drug susceptibility or resistance? Various traditional methods have been applied. The main strategies to detect AMR are based on techniques such as cell culturing, polymerase chain reaction (PCR), enzyme-linked immunosorbent assay (ELISA), spectrofluorimetry, and matrix-assisted laser desorption/ionization time-of-flight (MALDI-TOF) mass spectrometry [3,16,17,18]. On the other hand, cancer biomarker tests [19], positron emission tomography (PET) [20], and fresh tumor culture toxicity assays have been used to detect CMR in vitro and in vivo [21]. Although these methods have been successfully demonstrated, some important drawbacks should be cited, such as their time-consuming experimental steps, tedious sample preparation procedures, high cost, need for bulky instrumentation, and well-trained operators to be performed [22]. These disadvantages often limit their use in rural and remote regions and make them unsuitable for rapid diagnostics [18]. The use of alternative methods and techniques that can overcome these shortcomings is crucial in the fight against drug resistance.

Electrochemical methods have attracted considerable attention for bioanalytical applications because of their high sensitivity, operational simplicity, and ability to quickly provide accurate results, often within seconds [23]. Electrochemical techniques and sensors can provide a suitable route for the analysis of complex samples, such as blood and urine, and do not require lengthy sample preparation steps [24]. These analytical tools have the potential to be miniaturized and integrated into portable devices for point-of-care (POC) applications, including clinics and hospitals [25]. Such advantages make these methods highly attractive for the development of assays that can rapidly detect AMR and CMR in clinical samples. Several research papers describing electrochemical approaches to detect AMR and CMR have been published, employing techniques such as cyclic voltammetry (CV) [26], differential pulse voltammetry (DPV) [27,28,29,30], square wave voltammetry (SWV) [31,32,33,34], electrical impedance (EIS) [35,36,37], and scanning electrochemical microscopy (SECM) [23,38,39,40]. However, the electrochemical detection of AMR and CMR is not commercially available. While signal origin and sensitivity remain the most pressing points to improve, electrochemical methods show great promise to advance current detection technologies and benefit patients and healthcare systems around the world.

**Scheme 1:**
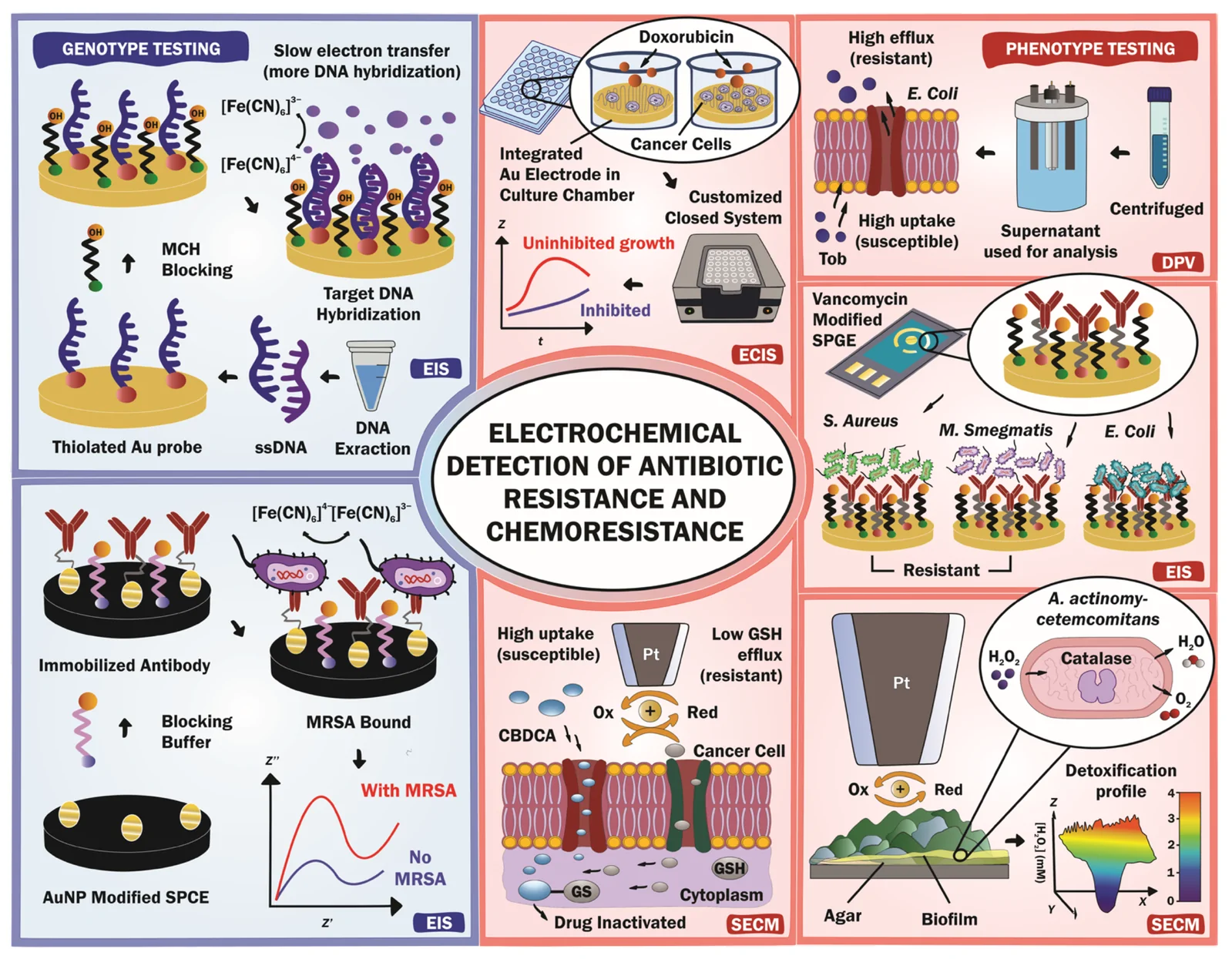
Scheme presenting major research strategies applied for the electrochemical detection of AMR and CMR.

This review presents recent reports of electroanalytical methodologies with the potential to be integrated into devices to clinically detect AMR and CMR. Initially, traditional techniques for investigating resistance are described, followed by a discussion about the application of electrochemical methods for detecting antibiotics and chemotherapeutics as well as quantifying resistance. State-of-the-art electrochemical detection of drug resistance can be roughly segmented into two main divisions: methods based on genomic assays or those based on cellular activity. The former involves the detection of genetic elements corresponding to drug resistance, whereas the latter correlates cellular activity to a specific resistance phenotype (Scheme 1). This review explores the strengths, limitations, and suitability of genomic or cell activity-based methodologies for the quantification of drug resistance in laboratory and clinical samples. While both strategies can be used independently to broadly identify AMR and CMR, they are also complementary when aiming to develop a comprehensive understanding of resistance. Genomic methods can be used to screen for resistance-related genes regardless of their expression, or lack thereof, under certain experimental conditions. On the other hand, activity-based methods can be used to elucidate details regarding the underlying resistance mechanism. Lastly, biosensor design strategies and POC devices to detect AMR and CMR are discussed, as well as current challenges and perspectives regarding the commercialization and widespread utilization of electrochemical assays to detect drug resistance in healthcare facilities worldwide.

## 2. Traditional Method of Detection

It is important to first understand the insurmountable limitations of traditional methods. Many traditional methods applied to investigate drug resistance include cell culture, polymerase chain reaction (PCR), enzyme-linked immunosorbent assays (ELISA), and various imaging techniques like positron emission tomography (PET). These techniques have been the gold standard for many years and are widely used in clinical and research settings.

For instance, culturing cells or bacteria can take days or weeks, which delays diagnosis and treatment decisions. The process also requires skilled technicians, involving careful handling, monitoring, and sophisticated knowledge of data interpretation [16]. In addition, risks for false-positives/negatives are significant, potentially further delaying a diagnosis. Notably, in vitro conditions may not always accurately replicate the in vivo environment of pathogens, potentially leading to discrepancies in result outcomes. Importantly, techniques such as PCR require expensive equipment and reagents, which may not be readily available where testing is needed, such as remote settings. In addition, PCR can only detect resistance genes that are already known and targeted by the assay, potentially missing mutations [17]. While ELISA is faster than cell culture, it still requires several hours for analysis. Furthermore, ELISA can only detect proteins and cannot be used to identify resistance at the genetic level. Imaging techniques, too, are typically expensive and require sophisticated equipment and facilities that might not always be available. While imaging provides a comprehensive view, it often lacks the resolution needed to detect small or early-stage resistant cells or bacteria. Techniques like PET involve exposure to radiation, which poses risks, especially with repeated scans [20].

It cannot be denied that the above traditional methods have many advantages, such as accuracy and reliability; however, the above-mentioned disadvantages cannot be ignored. These limitations drive the search for alternative, cost-efficient, portable, and accessible technology, such as electrochemical sensors, which aim to provide rapid and economic sample assessment for drug susceptibility or resistance.

## 3. Electrochemical Sensing of Antibiotics and Chemotherapeutics

An electrochemical sensor is a device that can quantitatively or qualitatively detect a biologically relevant analyte of interest based on its electrochemical properties. The detection principle exploits redox reactions of the analyte on the sensor’s electroactive surface to generate a signal that is converted to a measurable response, such as current, which is directly proportional to the concentration of the target substance [41]. Electrochemical sensors are highly versatile and are fabricated from a variety of conductive materials including gold, platinum, and carbon allotropes, such as graphite, carbon black, and glassy carbon [42]. Sensor fabrication can be achieved using different techniques, such as screen-printing [43]. The detection performance of electrochemical sensors can be improved through the modification of the electroactive surface using a range of materials and (bio)molecules, which increases the method’s sensitivity, selectivity, and specificity [44]. Flexible composition, geometry, and surface functionalization are particularly important for medical applications, since they enable the fabrication of ultrasensitive biocompatible detection platforms that can be fully integrated into portable electrochemical detection systems. As a result, electrochemical sensors are receiving considerable attention from researchers worldwide for the quantification and detection of drugs in environmental, biological, and pharmaceutical formulations.

While traditional methods often look for genomic information, our research has taken a different approach over the last decade. Instead of looking for gene expression, which might not always reflect a resistant phenotype of an organism depending on the environmental conditions, we are aiming to detect cellular resistance activity. While the overall manuscript explores literature concerning both strategies, studies from our research group report on the interaction of cells with antibiotics, antimicrobials, and chemotherapeutics.

One of the main steps toward developing electrochemical sensors capable of detecting AMR and CMR activity is the comprehensive understanding of the redox properties of antibiotics and chemotherapeutics. The chemical structure of many pharmaceutical compounds contains functional groups that can be oxidized and/or reduced at relatively mild electrode potentials, such as phenol and amino substituents, allowing the characterization and detection by electrochemical techniques. A complete understanding of the redox properties of any therapeutic agent should include the identification of oxidation and reduction potentials, the study of the molecule’s adsorptive or diffusive behavior, and the investigation of the influence of the supporting electrolyte’s pH and composition on the redox properties of the drug. To this end, many antibiotics and chemotherapeutics have been recently characterized using electrochemical methods and diverse electrode materials (Table S1) [28,29,30,31,32,34,45,46,47,48,49,50,51,52,53,54,55,56,57,58,59,60,61,62,63,64,65,66,67,68,69,70,71,72,73,74,75,76,77,78,79,80,81,82,83,84,85,86]. Our team published a review paper in 2020 [23], presenting a comprehensive description and discussion on the electrochemical detection of pharmaceuticals whose therapeutic effects are known to be affected by drug resistance mechanisms in bacterial and cancer cells. Recently, several reports on this subject have emerged [46,52,57,70,73,82], and selected examples will be discussed in the next section to highlight key elements of successful electrochemical sensors: reasonable detection ranges, simplicity, and analysis of complex media.

### 3.1. Antibiotic Detection

**Figure 1:**
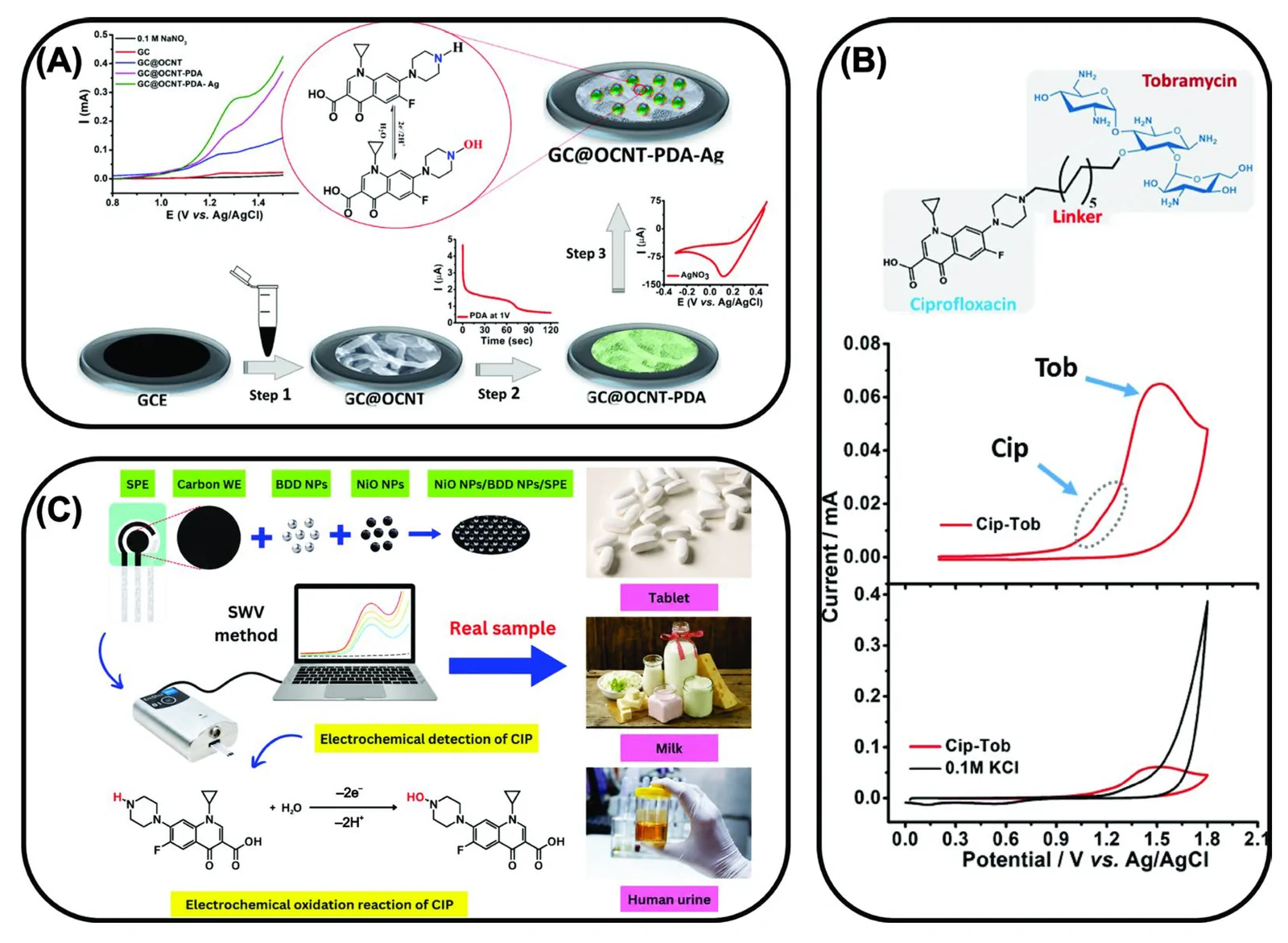
(**A**) Schematic illustration of the steps reported by Singh et al. [31] to obtain a conductive composite consisting of oxygen-functionalized carbon nanotubes, polydopamine, and electrodeposited silver nanoparticles (OCNTs-PDA-Ag) at a GCE to electrochemically detect ciprofloxacin. (**B**) The fabrication, application, and associated electrochemical oxidation of ciprofloxacin for the NiO and BDD NP modified SPE reported by Sutanto et al. [88]. (**C**) Electrochemical oxidation of ciprofloxacin-tobramycin hybrid (Cip-Tob) during CV at 50 mV s^−1^ in 0.1 M KCl, as reported by Islam et al. [26]. Figures obtained with permission from references listed above.

A useful example is our recent work that developed a promising voltammetric sensor to detect the antibiotic ciprofloxacin (Cip) [31], a broad-spectrum fluoroquinolone used to treat a number of bacterial infections in both humans and animals. The excessive use of this drug to treat even minor infections has resulted in the emergence of resistance mechanisms in pathogens, which significantly decreased its effectiveness [87]. The sensor utilized glassy carbon electrodes (GCEs) modified with a conductive composite consisting of oxygen-functionalized carbon nanotubes, polydopamine, and electro-deposited silver nanoparticles (OCNTs-PDA-Ag) (Figure 1A). Cip was characterized using cyclic and linear sweep voltammetry techniques (CV and LSV, respectively), showing an oxidation peak that was not significantly affected by pH changes. The presence of the conductive composite enabled this sensor to detect Cip at concentrations as low as 750 pM in tap water samples using SWV, with no significant interference from metal ions and other drugs, such as ampicillin and tobramycin (Tob). The high analytical sensitivity of this electrochemical sensor is a direct consequence of the presence of a nanocomposite consisting of carbon nanotubes, poly(dopamine) (PDA) and silver nanoparticles. Carbon nanotubes present high electrical conductivity, high surface area, and the presence of oxygen-containing groups that favor the interaction with the polar groups in the Cip molecules. Highly conductive silver nanoparticles and the polar conductive polymer PDA further enhanced the sensing properties of carbon nanotubes, creating a nanocomposite with superior physicochemical properties enabling the detection of Cip at picomolar detection levels. This electroanalytical method is promising for drug susceptibility studies, since the consumption of water containing (even trace) concentrations of antibiotics is a factor that contributes to the spread of AMR.

Subsequent research on Cip detection has focused on translating high analytical sensitivity into cost-effective, portable, and green sensing platforms suitable for on-site use. In this perspective, Sutanto et al. developed an innovative electrochemical sensor using disposable screen-printed electrodes (SPEs) modified with boron-doped diamond nanoparticles (BDD NPs) and nickel oxide nanoparticles (NiO NPs) to monitor Cip [88]. This study demonstrates substantial progress in simple and sustainable green electrochemistry since the electrocatalytic NiO NPs were synthesized through an eco-friendly protocol using *Spatholobus littoralis Hassk* root extract as a reducing and stabilizing agent. Using SWV, this portable platform offered a rapid detection response and achieved a limit of detection (LOD) of 0.054 µM over a linear concentration range of 0.1 to 100 µM. The simple drop-casted, disposable SPEs in this study represent crucial progress, which was validated for Cip monitoring in complex real matrices such as commercial tablets, cow’s milk, and human urine (Figure 1B).

In an analogous study, Yossief conducted the electrochemical characterization and determination of auranofin, a gold-based drug used mainly to treat rheumatoid arthritis, but that has been shown to be a promising agent to treat infections caused by bacteria resistant to traditional antibiotics [32]. GCEs were coated with a layer of carbon black to increase the sensitivity towards auranofin. Upon conducting CV, two anodic and two cathodic peaks were observed, which were ascribed to an adsorptive redox process that showed to be independent from the medium pH. This indicates that auranofin could be electrochemically detected in environmental and physiological samples in a broad pH range. Our study reported detection and quantification limits of 150 nM and 200 nM, respectively, using LSV. This was the first time that auranofin was studied by electrochemistry, and this sensitive approach is promising for the development of rapid drug susceptibility assays for clinically relevant bacteria.

Beyond the detection of single antibiotic targets, our group has explored the electrochemistry of hybrid antibiotics, synthetic molecules composed of two or more antibiotic molecules covalently bound. These hybrid compounds have been used as a strategy to overcome AMR, since each antibiotic moiety in the resulting hybrid molecule contributes one mode of action [89]. For the first time, Islam et al. [26] characterized a hybrid antibiotic, ciprofloxacin-tobramycin (Cip-Tob), using an unmodified GCE as working electrode. Cyclic voltammograms showed two anodic peaks, which corresponded to the individual oxidations of Cip and Tob (Figure 1C). Both oxidations were found to be independent of pH and were controlled by adsorption. The developed methodology resulted in detection and quantification limits for the hybrid drug, which were equal to 7 and 250 μM, respectively. Hybrid Cip-Tob has been shown to be an effective treatment against wild-type and drug-resistant *Pseudomonas aeruginosa* isolates [90]. The electroanalytical method described above could be used to quickly evaluate the uptake of this new drug by other resistant bacteria of concern.

While unmodified electrodes are most desirable when aiming for a commercial sensor, because of their simplicity, surface modifications using molecularly imprinted polymers (MIPs) offer significant progress in developing electrochemical platforms for sensitive and rapid antibiotic detection in complex real-world samples [91,92,93]. Recent work has established MIP-based sensors as robust, biomolecule-free tools for monitoring antibiotic residues. In 2026, Singh et al. used electrochemical techniques to synthesize a polypyrrole MIP for colistin (COL) detection on a screen-printed gold electrode (MIP/Ppy/AuSPE), and monitored COL rebinding by CV, DPV, and EIS [91]. The sensor detected COL from 0.001 to 100 µg mL^−1^ in milk with a LOD of 0.23 ng mL^−1^ after 10 min and recoveries of 87.5–93.9% in spiked samples. These results show that a disposable, fully synthetic MIP electrode can deliver laboratory-grade sensitivity and stability in untreated dairy matrices, demonstrating its suitability for real-time antibiotic detection in healthcare, agriculture, and food safety applications.

Other studies apply similar MIP-based strategies to the aminoglycoside tobramycin (TOB), an antibiotic widely used for treating serious Gram-negative and ocular infections but is increasingly compromised by emerging resistance in pathogens like *P. aeruginosa* [94]. War et al. fabricated a polyaniline-silver nanoparticle MIP on a gold screen-printed electrode and integrated it with a voltametric electronic tongue [92]. This platform detected TOB from 0.001 to 60 pg mL^−1^ with a DPV LOD of 1.9 pg mL^−1^ after 30 min and gave quantitative recoveries in chicken, beef, turkey, etc. Furthermore, chemometric analysis distinguished contaminated milk samples, demonstrating this platform’s potential as a practical screening tool for routine food safety monitoring. The work of Jamshid et al. further increased sensitivity by electropolymerizing a poly(1,5-diaminonaphthalene)-zinc oxide MIP on an Au/reduced-graphene-oxide substrate (MIPN-8), reaching a 4.2 fM detection limit over 10^−12^–10^−16^ M after 150 s with high selectivity versus gentamicin (GEN) and kanamycin (KAN) [93]. Moreover, with 98–105% recoveries in eye drops, eggs, and milk, this platform becomes directly applicable to quality control, residue monitoring, and therapeutic drug monitoring in environmental, agricultural, and pharmaceutical sectors.

The requirements are clear: electrochemical sensors for antibiotics, antimicrobials, and hybrid molecules must deliver sensitive assays, reaching LODs in the femto- to micro-molar range, must be affordable to produce, and need to enable access to complex matrices, such as urine, blood, and food samples.

### 3.2. Chemotherapeutic Detection

The electrochemical detection of chemotherapeutics has been increasingly reported in the literature, including drugs whose efficacy has been threatened by CMR. A typical example is the drug 5-fluorouracil (5-FU), which has long been used to treat various types of tumors, including colorectal, stomach, breast, and cervical ones. This uracil analog was detected electrochemically by Bojko et al. [33] using a carbon paste electrode modified with porphyran-capped silver nanoparticles. The voltammetric analysis of 5-FU revealed an oxidation peak that was influenced by the electrolyte’s pH. The presence of the nanoparticles allowed this sensor to detect 5-FU in concentrations as low as 10.7 μM by SWV, which enabled its application to accurately detect the drug in real pharmaceutical formulations. This study is an example of a straightforward electroanalytical methodology with potential to be utilized in the quality control of pharmaceutical products containing 5-FU. Methods to provide a precise and accurate quality control of 5-FU formulations can aid in the minimization of chemoresistance, since they prevent administration of inadequate dosages to the patients, thereby reducing CMR development.

Shojaei et al. reported an electrochemical sensor to detect the chemotherapeutic imatinib (IMA), an oral tyrosine kinase inhibitor used to treat different types of leukemia and gastrointestinal stromal tumors [81]. The sensor was based on the simultaneous modification of carbon paste electrodes with three substrates: multiwalled carbon nanotubes (MWCNTs), Au@Fe_3_O_4_ nanoparticles, and an IMA-specific MIP. The nanomaterials conferred to the sensor an enhanced electrical conductivity and maximized surface area, while the MIP increased selectivity. IMA was fully characterized by CV, showing a diffusion-controlled oxidation process that was dependent on the pH. This interesting sensing platform detected IMA by DPV in a broad concentration range, 1.0–1000.0 µM, with a detection limit of 13 nM. The effectiveness of the proposed sensor in complex matrices was investigated and confirmed by determining IMA in tablet and serum samples. Moreover, using a simpler, low-cost recognition scheme, the recent work of Roushani et al. [95] advances the field by showing that a strongly electrocatalytic nanocomposite can reach much lower detection limits for a chemotherapeutic crizotinib without relying on a molecularly imprinted layer. They did this by modifying a GCE with a nanocomposite made of two parts: a NiCoCu-layered triple hydroxide (LTH) and MWCNTs, which maximized the surface area for crizotinib oxidation. Under optimized CV and DPV conditions, the modified NiCoCu-LTH/MWCNT/GCE sensor gave a linear response from 0.001 to 1500 nM with an ultralow LOD of 0.0003 nM, demonstrating good selectivity and reproducibility. Both studies gave results that did not differ statistically from high-performance liquid chromatography (HPLC). This research illustrates the advantages of utilizing MIPs and nanomaterials for the fabrication of electrochemical sensors to detect chemotherapeutics, which enables the detection of drug molecules at trace concentrations with high selectivity, even in complex biological samples.

Using a simpler sensor consisting of a glassy carbon electrode (GCE) modified with graphene sheets decorated with electrodeposited bismuth nanoparticles (BiNPs), Khumngern et al. [78] recently developed an electroanalytical method to detect the well-known anticancer drug cisplatin. This compound is usually effective at treating several tumor types at the beginning of chemotherapy, but patient responses frequently decrease over time because of drug resistance development [96,97]. The BiNPs electro-catalyzed the diffusion-controlled oxidation process of cisplatin, significantly enhancing the electrochemical anodic current. After a complete electrode optimization, cisplatin was detected in the range of 6.0–180 µM using DPV, with detection and quantification limits equal to 4.4 and 14.5 µM, respectively. In this study, BiNPs were chosen due to their electrocatalytic effect on the oxidation of cisplatin, in addition to an increase in electrochemically active surface area. The demonstrated high sensitivity and selectivity of this sensor enabled the analyte detection in human serum samples, showing results that correlated with those obtained by HPLC. This sensor shows that understanding the electrochemical behavior of target drugs is crucial for the development of efficient electroanalytical methodologies and sensors.

To advance electrochemical detection by coupling it with spectroscopy, Khan et al. [77] recently proposed a dual-signal spectro-electrochemical sensor for gemcitabine (GMT), an anticancer drug that promotes cell death by blocking DNA replication. The core element of this sensor was a GMT-specific polypyrrole-based MIP. The further binding of bovine serum albumin-Ag nanoparticles nanoprobes (BSA-AgNPs) to the GMT molecules captured by the sensor had the role of enhancing the drug’s oxidation current. UV-Vis detection was performed by quantifying the BSA-AgNPs nanoprobes that did not bind to the immobilized GMT molecules on the sensor surface. After full optimization and thorough characterization, this spectro-electrochemical assay could detect GMT with high specificity in the concentration ranges of 1.0–200.0 μM and 0.5–200.0 μM for the spectroscopic and electrochemical detection modes, achieving quantification and detection limits of 0.4 μM and 0.15 μM, respectively. The fabricated sensor was able to successfully differentiate serum samples from lung cancer patients and healthy volunteers regarding the presence of GMT, with results that were validated by liquid chromatography–mass spectrometry (LC-MS). This interesting sensing strategy can be ideally adapted to detect other chemotherapeutics presenting affinity to BSA simply by changing the drug molecules during the electrochemical synthesis of the MIP. The dual detection mode confers an additional confirmation for electrochemical analysis, and could be applied, for example, in drug uptake studies with living cancer cells.

Building on this work, more recent studies have focused on simplifying sensing platforms while retaining high sensitivity and selectivity. Specifically, Harijan et al. developed a molecularly imprinted polypyrrole film on a GCE by electropolymerizing pyrrole in the presence of 5-FU, then using a voltammetric scan to remove the embedded drug and expose recognition sites [98]. The resulting MIP sensor was characterized by CV, DPV, and EIS and provided a linear response between 2 and 42 µM with a LOD of 0.605 µM. More importantly, fabrication relies on a single electrochemical step and avoids extraction using organic solvents, which reduces cost and complexity compared to conventional analytical workflows. This study shows that the MIP sensor operates effectively in pharmaceutical and biological matrices, demonstrating its potential to support on-site therapeutic drug monitoring in oncology clinics where maintaining 5-FU within a narrow therapeutic window is critical to avoid dose-limiting toxicity and chronic under-exposure, both of which can drive treatment failure and chemoresistance [99].

Works discussed above are successful examples for the direct recognition and quantification of antimicrobials and chemotherapeutics at electro-active surfaces, opening the door for electrochemical drug susceptibility testing. Furthermore, they clearly present the importance of understanding the electrochemical behavior of antibiotic and chemotherapeutic compounds, as well as the usefulness of functionalized electrode surfaces.

Electrode fouling, interference, and adsorption are also major obstacles for many different biosensors. This is attributed to the wide range of organic compounds, large biomolecules, and even cells that can adsorb onto electrode surfaces and contribute to the electrochemical background noise through redox processes with the electrode. Various strategies have been employed to address these problems, such as neutral or zwitterionic polymer [100,101], peptide [102], or composite-based [103] electrode coatings. Marin et al. recently demonstrated a polyethylene glycol (PEG) monolayer to reduce non-specific adsorption for label-free immunosensing [101]. One particularly interesting aspect of their strategy was the incorporation of covalently bound ferrocene moieties into the PEG chains to promote electron transfer. This is important since one of the drawbacks of coating electrodes with antifouling layers is that it may impede diffusion of the analyte, thereby reducing the sensitivity of the biosensor. Sanooba and Alwarappan utilized a zwitterionic poly(sulfobetaine methacrylate) coating to reduce fouling for the detection of uric acid and dopamine [100]. While the authors report that the coating imparts excellent electrical conductivity and hinders the adsorption of oxidation products onto the electrode surface, they caution that large biomolecules may still adsorb to the surface in more complex clinical samples. A recent example of peptide-based coatings was demonstrated by Song et al. [102] to hinder the adsorption of biomolecules. The zwitterionic nature of their coating encouraged the formation of a dense hydration layer that imbued the sensor with excellent antibiofouling properties. Moreover, it afforded structural rigidity and a degree of resistance to naturally occurring proteases. The authors report an impressive stability in real serum of three weeks. Wareham-Mathiassen et al. report a recent example of a nanocomposite coating for implantable biosensors [103]. This design utilizes a lattice matrix of BSA and reduced graphene oxide that is bound to both antibodies and an antibiotic. Although the authors report successful antimicrobial and antifouling properties, good electrical conductivity, and validation as a sensitive immunosensor, the biosensor is only stable for three weeks, which is not sufficient for an implantable biosensor. While these examples aim to introduce the reader to the issue of electrode fouling and demonstrate selected examples of state-of-the-art strategies to combat it, we suggest consideration of comprehensive reviews on these topics [104,105].

## 4. Investigation of Drug Susceptibility by Electrochemistry

To assess the efficacy of drugs towards bacteria and cancer, drug susceptibility tests are crucial as they provide guidance to physicians to find drugs and optimum dosages that will be efficient to treat patients on an individual basis [18]. In hospitals, individuals affected by a resistant disease are often treated empirically with medication, and this exposure contributes to the rise in AMR and CMR worldwide [106].

Due to their high sensitivity, rapid response, and compatibility with miniaturized platforms, electrochemical methods offer significant advantages for the development of drug susceptibility tests to detect AMR and CMR directly in patient samples [23]. Such approaches have the potential to substantially improve clinical diagnostics by overcoming the limitations of current susceptibility testing methods, which are often complex, expensive, and time-consuming [107]. Consequently, considerable research efforts have focused on developing electrochemical proof-of-concept platforms and detection strategies for AMR and CMR [37,108,109,110,111,112]. Electrochemical (bio)sensors have been employed to assess drug susceptibility either by directly detecting drug molecules consumed or expelled by bacteria or tumor cells, or by measuring resistance-associated biomarkers released by these cells, including specific proteins and metabolites. The following subsections provide an overview of recent electrochemical approaches for detecting drug resistance in bacteria and cancer (Table 1).

### 4.1. AMR Detection

**Figure 2:**
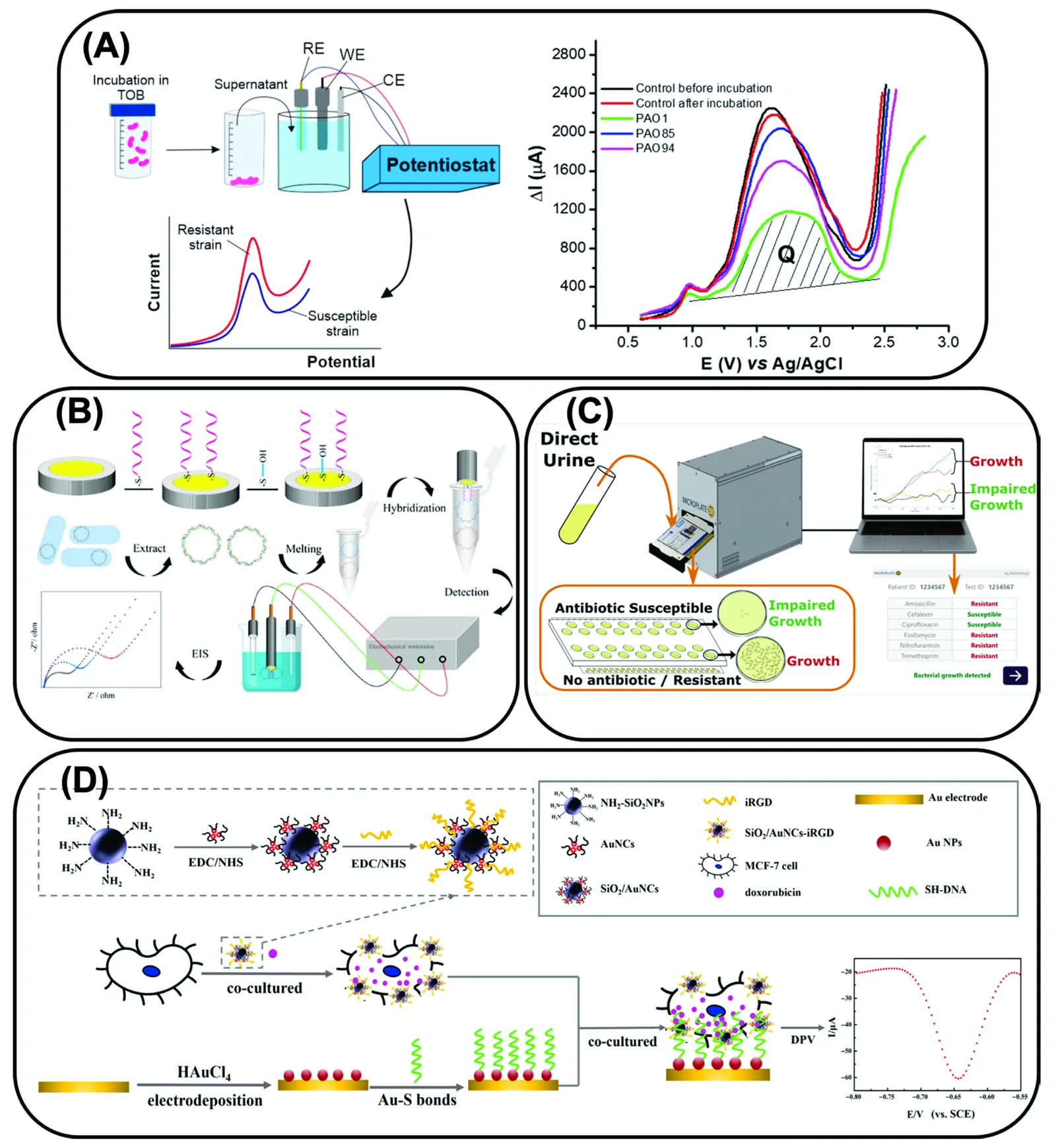
Schematic representations of (**A**) the electrochemical assay developed by Lopes et al. [27] to perform Tob retention studies with susceptible and resistant *P. aeruginosa* strains, and differential pulse voltammograms of Tob solutions after a 15 min incubation in the presence of each strain. (**B**) Modification of a thin-film gold electrode with thiolated single-stranded DNA probes for the detection of antibiotic resistance genes by EIS as reported by Wan et al. [120]. (**C**) Schematic diagram of the Dx Rapidplate microelectrode array system to monitor AMR in bacteria in untreated urine samples described by Ralston et al. [85]. (**D**) The fabrication and application of SiO_2_/AuNCs-iRGD NPs for the monitoring of doxorubicin-associated AMR in MCF-7 cells described by Yu et al. [125]. Permissions for figures obtained from references listed above.

Many electrochemical techniques for detecting AMR rely on genomic assays or quantification of biomarkers. However, one of the approaches that distinguishes our work from others is that our methods quantify cellular activity rather than genomic information to differentiate between resistant and susceptible phenotypes. This has the advantage that we can detect the actual susceptible/resistant behavior of cells, which includes mutations or activation and inactivation of genes that might occur as a result of treatment, which otherwise would not be detected. We reported a straightforward and rapid electrochemical methodology to detect AMR in susceptible and resistant *Pseudomonas aeruginosa* cells [27]. Lung infections caused by resistant strains of this Gram-negative bacterium are a serious threat to immunocompromised patients, considerably increasing the mortality rate among cystic fibrosis patients [113]. *Pseudomonas aeruginosa* has the ability to alter and modulate the human immune response and can be intrinsically resistant to antibiotics [114], including the aminoglycoside tobramycin (Tob). Lopes et al. [27] used an unmodified GCE to quantify Tob retention in susceptible and resistant *P. aeruginosa* strains. Firstly, our team electrochemically characterized Tob using CV and DPV measurements and found that this antibiotic was irreversibly oxidized on the GCE surface through an adsorption-controlled process. Tob showed an electrochemical response in pHs ranging from 1.0 to 9.0, which enables its determination in samples across this broad range of pH conditions. A maximum current was observed at pH = 1.0, which was chosen for subsequent analysis. In the second part of our study, *P. aeruginosa* strains with different degrees of resistance to Tob were analyzed using DPV. PAO1 is a wild-type susceptible strain, whereas PAO85 and PAO94 are Tob-resistant strains, which overexpress MexXY-OprM membrane efflux pumps. Additionally, PAO85 is a mucoid strain that can synthesize several virulence factors and large amounts of alginate exopolysaccharide, which allows it to form biofilm-protected microcolonies, therefore increasing their resistance to the action of antibiotics. The different strains were incubated in a 2 mM Tob solution for 15 min at 37 °C, centrifuged, and the supernatants were analyzed using DPV as depicted in Figure 2A. PAO1 induced a pronounced decrease in Tob anodic current because of its consumption by the cells. On the other hand, PAO85 and PAO94 induced less significant decreases in current, which was ascribed to their resistant phenotype. Interestingly, the higher electrochemical signals exhibited by PAO85 compared to PAO94 clearly show that the reported approach not only distinguishes between Tob-susceptible and Tob-resistant bacteria, but it was also able to differentiate strains presenting different degrees of resistance towards Tob. This study provided a solid foundation for future quantitative drug efflux and retention studies in more complex patient samples. It is important to emphasize that this method does not require complex and costly electrode modification steps, and was able to detect AMR in living *P. aeruginosa* cells in only 15 min. Further details regarding resistance quantification in *pseudomonas* can be found in the 2024 review from our research group [115].

In a more recent 2024 study [116], our group extended the same electrochemical approach from *P. aeruginosa* to *E. coli* to probe Tob susceptibility across species. In this study, Lopes et al. [116] re-optimized Tob oxidation by varying electrode material, pH, and DPV parameters, achieving a two-fold increase in signal sensitivity in comparison to the *P. aeruginosa* assay on unmodified GCE [27]. They then incubated two susceptible strains (*E. coli* ATCC 25922 and EC 94393) and one resistant clinical isolate (EC94474) in 2 mM Tob for short periods. Using the optimized DPV parameters, the drug remaining in the supernatant and the fraction taken up by cells was quantified and tracked over time. When they followed the resistant isolate over shorter intervals, they saw an initial drop in current due to Tob uptake, followed by recovery of the current between 5 and 15 min. This indicated that Tob was being expelled back into the medium and regenerating the electrochemical signal rather than being retained inside cells. This time-dependent decrease and subsequent recovery of the Tob oxidation current for EC94474 contrasted with the sustained low signal for ATCC 25922 and EC 94393, demonstrating that *E. coli* uses an efflux pump-mediated resistance mechanism instead of uptake inhibition. This study validates the retention assay in another clinically important Gram-negative species and shows that the same electrochemical platform can detect both efflux- and uptake-related resistance mechanisms. In fact, our team published a review paper in 2024 [117], which stresses the importance of rapid, phenotypic antimicrobial susceptibility testing (AST) methods that capture real-time drug interactions with cells, which can be suitable for point-of-care use in managing multidrug-resistant Gram-negative infections. Our work on *P. aeruginosa* and *E. coli* directly addresses the needs highlighted in this review.

Methods developed in our laboratory up to this date primarily employ unmodified electrode surfaces; however, the modification of conducting surfaces with biomolecules such as nucleic acids, aptamers, and antibodies has been a common approach for the electrochemical detection of drug resistance in bacteria [46,115,118]. The use of such biomolecules is particularly interesting due to their high specificity to recognize and bind their targets, which results in highly selective biosensing tools [119]. This strategy has been used extensively for the genomic detection of antibiotic-resistant genes (ARGs). Wan et al. have recently reported an interesting electrochemical biosensor to detect bacterial ARGs in water samples [120] (Figure 2B). ARGs are usually located in plasmids or transposons and can be transferred from cell to cell, allowing the resistance to rapidly spread throughout a bacterial population [121]. Initially, ARGs encoding the enzyme β-lactam hydrolase were incorporated into *E. coli* by transformation, which made them resistant to β-lactam antibiotics, such as ampicillin. Afterwards, the authors modified gold disk electrodes with a thiolated DNA probe that binds specifically to the ampicillin resistance gene (ARG_AMP_), followed by treatment with 6-mercapto-1-hexanol (MCH) to block the empty sites of the electrode. The biosensor’s detection principle was based on the hybridization of the immobilized DNA probe with the ARG_AMP_ after its extraction from the resistant *E. coli* cells and denaturation. The hybridization occurrence was indicated by increases in the charge transfer resistance (R_ct_) of the system, which were obtained by EIS analysis in the presence of [Fe(CN)_6_]^3−^ ions as a redox indicator. After fully optimizing the DNA probe concentration, incubation times, and hybridization temperature, the authors were able to detect the ARG_AMP_ in the range of 6.3 to 900.0 ng mL^−1^, with good reproducibility and specificity. With an assay time of 1 h, this efficient biosensing platform demonstrates its potential to detect ARGs in water samples, in which ARGs can be commonly found.

Further, taking advantage of the high binding affinity and specificity of antibodies towards their targets, Khue et al. developed an electrochemical immunosensor to detect methicillin-resistant *Staphylococcus aureus* cells [122]. Such strains are one of the main causes of hospital-acquired infections, and therefore their detection is extremely relevant to health care systems worldwide. Disposable SPCEs were modified with gold nanoparticles (AuNPs) that were functionalized with antibodies that specifically recognize the resistant pathogen of interest. The AuNPs were incorporated in the biosensing platform due to their high electrical conductivity and biocompatibility, which enhances the electron transfer rates while maximizing the load of antibody molecules immobilized at the electrode. Using R_ct_ responses from EIS measurements in the presence of [Fe(CN)_6_]^3−^/[Fe(CN)_6_]^4−^ ions, the proposed immunosensor efficiently detected the resistant *S. aureus* cells at concentrations ranging from 10 to 10^6^ CFU mL^−1^, with an assay time of only 30 min. Importantly, this biosensor did not recognize *E. coli* cells, which confirms the high binding specificity of the immobilized antibodies. While electrodes modified with biorecognition elements demonstrate impressive results, they are more costly and are incapable of continuous monitoring, as the binding and unbinding kinetics are often sluggish for surface-bound biorecognition elements such as antibodies and aptamers. Furthermore, such genomic or antigen-based assays only screen for *known* markers, whereas our activity-based assays can investigate the resistance phenotype of a given cell line without deep understanding of the corresponding genome or biochemical composition.

Whereas the studies described previously were focused on genomic assays or detecting electroactive antibiotic molecules retained or expelled by susceptible and resistant bacteria, the research by Dizaji et al. [36] aimed to detect whole susceptible bacterial cells on an electrochemical biosensing platform. Commercially available screen-printed gold electrodes (SPGEs) were covalently modified with the antibiotic vancomycin (Van), which was previously functionalized with thiol groups. The biosensor selectively recognized and captured susceptible Gram-positive bacteria in whole-cell form, followed by EIS analysis in the presence of [Fe(CN)_6_]^3−^/[Fe(CN)_6_]^4−^ redox mediator. The binding of susceptible bacteria was indicated by pronounced variations in the charge transfer resistance of the system. Vancomycin-susceptible *Staphylococcus aureus* cells were successfully detected using the proposed biosensor due to their high affinity and strong attachment to the antibiotic-modified SPGE. On the other hand, resistant *Escherichia coli* and *Mycobacterium smegmatis* cells did bind significantly to the electrode. The electrochemical results were further validated by scanning electron microscopy (SEM) analysis, which confirmed that only *S. aureus* could be recognized by the biosensor, forming a dense monolayer on the electrode’s surface. The biosensor was also able to quantify *S. aureus* in the range of 10 to 10^8^ CFU mL^−1^, and detection and quantification limits were found to be equal to 10^1.58^ to 10^4.81^ CFU mL^−1^, respectively. This selective bioelectrochemical approach is an interesting example of a straightforward and low-cost method to detect susceptible bacteria and could be easily adapted to detect other types of susceptible pathogens in clinical samples in the future.

Recent work has remarkably advanced electrochemical AST from a proof-of-concept to real clinical deployment by validating an EIS-based platform on real, untreated urine samples from multiple hospitals [123]. In this study, Ralston et al. developed the Microplate Dx RapidPlate system, which uses gel- and redox-mediator-modified electrode arrays and EIS to monitor bacterial growth in the presence of antibiotics directly in urine, without pre-treatment [123] (Figure 2C). The system is implemented as a 32-channel cartridge and a reader that was evaluated using 89 clinical UTI samples across three sites in the U.K. and India. The performance was compared to standard AST methods such as VITEK 2 and disk diffusion. This platform successfully detected bacterial growth in 50 min and delivered antibiotic susceptibility profiles in 85 min. In fact, it achieved 95% concordance with the standard AST methods for common pathogens such as *E. coli* and *K. pneumoniae*. These findings are highly relevant in practice because they show that a low-cost, multiplexed electrochemical system can deliver reliable UTI susceptibility results in under 90 min, instead of the usual 24 to 72 h. Zhang et al. further advanced electrochemical AST toward decentralized use by integrating sensing and readout into a smartphone-based format for MRSA [124]. These platforms would directly reduce clinicians’ reliance on broad-spectrum antibiotics, hence limiting unnecessary antibiotic exposure [117]. This impedance-based method is similar to our work in the sense that phenotyping is not achieved by direct sensing of genes. While this study does not focus on cellular activity, but rather analysis of bacterial growth rates, the success and real-world applicability of this device is very encouraging for our own line of research.

### 4.2. CMR Detection

Electrochemical approaches to detect CMR in cancer have been based mainly on the use of unmodified metallic electrodes and sensors, including gold and platinum [23]. This contrasts with the electroanalytical methodologies to detect AMR, which are mostly based on the modification of conducting surfaces with nanomaterials and biomolecules. Instead of directly detecting cancer cells that have become resistant to medication, or resistance-related biomarkers, electrochemical methods to detect CMR have been mostly focused on monitoring drug consumption [126], and physicochemical cell parameters, such as pH and impedance [37,127].

In this perspective, our group has proposed the use of unmodified platinum electrodes to develop a voltammetric method to detect carboplatin (CBPt) consumption by susceptible (A2780-S) and resistant (A2780-CP) ovarian cancer cells [126]. Firstly, we conducted a full electrochemical characterization of CBPt, which showed to be oxidized irreversibly on the Pt surface according to a diffusion-controlled mechanism. This oxidation process was observed in a broad pH range (2.0 to 9.0), with the anodic potential being influenced by the pH in the range 5.0–9.0. The Pt electrode was able to detect CBPt concentrations ranging from 30 μM to 1 mM using LSV, and detection and quantification limits of 30 μM and 50 μM were achieved. To assess the consumption of CBPt by ovarian cancer cells by electrochemistry, different numbers of A2780-S cells were initially exposed to 1 mM CBPt in PBS for 40 min at 37 °C and 5% CO_2_. Cells were removed by centrifugation, and the supernatant was analyzed using LSV. The results consistently showed a decrease in the CBPt oxidation current with increasing cell number, suggesting the consumption of the drug by this susceptible cell line. Given that the developed electrochemical method could effectively detect drug consumption in living ovarian cancer cells, we next compared the CBPt consumption profile of A2780-S and A2780-CP cells. Although a sharp decrease in the oxidation current was observed in both cases, no significant differences were observed in drug consumption between the two cell lines. This was an indication that the chemoresistance mechanism in A2780-CP cells does not rely on the overexpression of membrane efflux pumps but rather must be based on intracellular factors that lead to the observed resistance phenotype. This study not only proved the efficiency of electrochemistry to quantify the consumption of an anticancer drug by living cancer cells but also generated insight about CBPt-resistance mechanisms.

While our work primarily utilizes voltammetric techniques such as DPV, SWV, and CV, as well as scanning electrochemical microscopy as discussed in Section 5 below, Fuentes-Vélez et al. designed an interesting customized system based on electric cell-substrate impedance sensing (ECIS) to detect chemoresistance in colon cancer [37]. The device was based on utilizing unmodified interdigitated gold electrodes conveniently placed at the bottom of a 48-well cell culture plate pre-coated with type I collagen. Wild-type colon cancer cells (HT-29-WT) and a variant resistant to the anticancer drug doxorubicin (HT-29-DX) were cultured in a plate suitably attached to the measuring device, and cell–substrate impedance was monitored over 140 h. All stages of adherent cell growth were evidently observed as variations in normalized impedance values: lag phase (cell adaptation and adhesion), log phase (exponential growth), plateau (contact inhibition), and cell detachment/death (due to confluence). Interestingly, a clear difference in the impedance curves was observed when comparing HT-29-WT and HT-29-DX cells, with the resistant variant showing a shorter plateau phase than their susceptible counterparts. This was attributed to the higher migratory tendency, higher spreading level, and different membrane composition of the resistant cells. In the presence of different concentrations of doxorubicin (administered after 48 h of cell seeding), the resistant cell line exhibited an impedance increase over 72 h that was more pronounced than the one observed for the susceptible cell line. Gene expression analysis proved that HT-29-DX cells presented a significantly higher expression of drug transporters, such as MRP1, MRP2, and ABCG2, which justifies their resistant characteristics. Besides clearly showing differences in the resistant phenotypes of cancer cells, the developed detection system could also be used to sensitively detect minimal doses of chemotherapeutics that present a cytotoxic effect on the cells. When compared to our group’s methods, this approach requires a longer analysis time (72–140 h) and might therefore not be immediately applicable to clinical settings in its current state. Nonetheless, it is an example that demonstrates that ECIS can be used as a sensitive and label-free method to monitor cancer development and progression in patients, as well as test drug effectiveness accurately. A significant advantage of this technique compared to our work is the ability to monitor various stages of cell growth, which could be useful for elucidating the details of how chemotherapeutics influence cancer cell growth.

In our laboratory, cells are investigated either on a 2-dimensional surface (Petri dish) or suspended in a buffer solution. This contrasts with recent studies which have shown that three-dimensional (3D) hydrogels represent a more accurate model of culturing cells in vitro when compared to flat surfaces (2D). In addition, 3D platforms exhibit mechano-metabolic similarities to in vivo tumor cells. A novel approach towards high-throughput and quantitative drug screening of cells cultured in the 3D environment was proposed by Lei et al. by combining the paper-based cell culture model and EIS technique to quantify the cellular response [127]. In this study, cancer cells were encapsulated and cultured in hydrogel to mimic the natural microenvironment of tumors. Two human hepatoma cell lines (Huh7 and Hep-G2) and two anticancer drugs (doxorubicin and etoposide) were used to demonstrate the impedimetric screening of drug sensitivity of cells. The authors demonstrated that cell viability could be quantified by EIS at 24 and 48 h, and the obtained results revealed that Huh7 cells had a higher drug resistance than Hep-G2 cells. Besides that, results indicated that doxorubicin had a higher efficacy compared to etoposide to treat hepatocellular carcinoma.

The latest work of Marino et al. further advances impedimetric screening methods by establishing a platform for continuous and real-time EIS measurements in complex 3D tumor cultures without requiring complex microfluidics [128]. A platinum multielectrode array is constructed where porous, dome-shaped scaffolds are fabricated directly on top of the measurement electrodes to anchor cancer cells into stable 3D spheroids. This study successfully showed that the platform records changes in impedance and cell index, which reflect structural alterations during tumor spheroid growth and dose-dependent drug responses. Most importantly, the EIS platform detects subtle cytotoxic effects within three hours of drug exposure, well before conventional optical imaging can capture physical damage to cancer cells. This study successfully demonstrates that this platform can accurately model drug susceptibility and chemoresistance in microscopic glioblastoma tumor cells that survive tumor removal surgery, directly addressing glioblastoma reoccurrence. In fact, this platform can serve as a promising high-throughput preclinical tool that accelerates the discovery of new chemotherapeutics and minimizes animal testing. While hydrogels are clearly useful for impedance-based drug resistance detection, the voltammetric techniques utilized in our research will require careful planning and consideration to successfully incorporate hydrogels. This is because our techniques are dependent on the diffusion of molecules to and from the electrode surface, which would be strongly influenced by the chemical and physical composition of the hydrogel. Thus, in order to extend hydrogel materials to our CMR research, a thorough understanding of the diffusion properties of redox-active molecules in hydrogels must first be obtained.

Similar to the previous AMR section, intriguing nanomaterial-modified sensors have been applied in CMR research, such as the recent findings from Yu et al., which advance chemoresistance detection by overcoming the cell membrane barrier to quantify intracellular drug accumulation directly [125]. Firstly, gold electrodes are modified through electrodeposition of AuNPs and incubation with thiolated DNA (SH-DNA). Then, they synthesized membrane-permeable composite nanoparticles made of three parts: silica nanospheres (SiO_2_), gold nanoclusters (AuNCs), and a circular transmembrane iRGD peptide (SiO_2_/AuNCs-iRGD). After incubation of SiO_2_/AuNCs-iRGD with MCF-7 breast cancer cells and doxorubicin (DOX), then with DNA-modified electrodes, the cell membrane permeability increased. This allowed the DNA strands that are bound to the electrode to safely enter the living cells and bind intracellular DOX through intercalation (Figure 2D). Using DPV, the electrons generated by the redox reactions from the intercalated DOX are then transferred along the DNA backbone to the electrode surface, where they are quantified. This platform operates effectively at physiological conditions without requiring cell lysis, achieving a broad linear detection range from 0.05 to 300 µmol/L and a low LOD of 0.01 µmol/L. The findings of this study can be pivotal to monitor trace levels of cellular drug uptake and adjust therapeutic dosages before chemoresistance occurs in clinical settings.

Approaches above highlight two intrinsically different signal generation modes. An on-signal is collected through the direct electrochemical conversion of a chemotherapeutic, as shown by our team [126], whereas EIS generates an off-signal [37,127]. The off-signal approaches are advantageous as they reveal drug susceptibility phenotypes independent of cellular resistance mechanisms. However, on-signal approaches offer selective and rapid analysis of samples, as the electrochemical signal is not limited by cell culture growth over time. On-signal detection assays for intracellular mechanisms exist and are based on sophisticated electrochemical methods, such as scanning electrochemical microscopy (SECM), as discussed in the next section.

**Table 1:** Recent promising investigations of electrochemical approaches to detect drug resistance in bacteria and cancer.

Bacteria/Cells	Drug/Compound	Sensor/Electrode	Electrochemical Technique	Detection Time	LOD	Reference
*P. aeruginosa* PAO1; PAO94 and PAO85	Tobramycin	GCE	DPV	15 min	0.34 mM	[27]
*S. gallinarum*	Ofloxacin	AuNPs/MWCNTs/SPCE	DPV	1 h	100 CFU/mL	[129]
	Penicillin					
*E. coli*	Gentamicin	R-SPEs	DPV	90 min	1.56 µM	[130]
*S. aureus*		AmAu/CPE	DPV	15 min	0.724 CFU/mL	[131]
*E. coli*					0.699 CFU/mL	
blood serum from	bevacizumab	Ab/DNA/GO/GCE	DPV		0.02 μg/mL	[86]
colorectal cancer patients’ volunteers						
*S. aureus*		SPGE-Van	EIS	1 h	101.58 CFU/mL	[36]
Methicillin-resistant	Methicillin	PEI-f-CNT	EIS		1.23 × 10^2^ CFU/mL	[108]
*S. aureus*						
*E. coli*	Streptomycin	SPGE	EIS	1 h		[132]
*E. coli*	Oxacillin	HRP-labeled dNTP/SPGEs	Chronoamperometry	60 min	319 cfus/mL	[112]
Methicillin-resistant	Cas12a enzyme	E-Si-CRISPR	SWV	90 min	3.5 fM	[133]
*S. aureus*						
*S. aureus*	Streptomycin, Oxytetracycline, Tetracycline and Ampicillin	GC hairpin aptamer/MCH/Kana/PBIB/FMMA	SWV	90 min	4 CFU/mL	[134]
*E. coli*					6 CFU/mL	
*B. subtilis*	Ciprofloxacin	l-CeONP/ITO	CV	15 min	9 × 10^10^ cfu/mL	[135]
*E. coli*	Cefixime, Amoxycillin				4.5 × 10^4^ cfu/mL	
MCF-7 cells	Adriamycin	PLL/MWCNT/GC	CV, SWV			[136]
Enteropathogenic *E. coli*	Nitrocefin	AuME-SAM-anti-EspB mAb-nitrocefin)	EIS, DPV	<3 h	EspB: 4.3 ng/mL β-lactamase: 3.6 ng/mL	[137]
Methicillin resistant *S. aureus*, *E. coli*	Penicillin, oxacillin, cefoxitin	SPCE/PBNC/BL-Ab with 3D hydrogel paper scaffold	CV, DPV, EIS	1–4 h	0.146 mU/mL; 10^2^ CFU/mL	[138]
Methicillin resistant and susceptible *S. aureus,*	Vancomycin	GR/SPE + VAN/BSA; AuNPs/MXene + anti-PBP2a	CV, DPV, EIS	2 h	5 CFU/mL	[139]
*E. coli*, *B. subtilis*	Ampicillin, tetracycline	SPCE in PDMS ε-µD; PLL	EIS	3 h	2 × 10^4^ CFU/mL	[140]
*E. coli*, *S. aureus*, *P. aeruginosa*	Cefotaxime, vancomycin	CTX-AuSPEs	EIS	18 h	6 × 10^2^ CFU/mL	[141]
*E. coli*, *P. aeruginosa, Salmonella typhimurium*, *Bacillus cereus*, *Listeria monocytogenes*	Ampicillin, gentamicin, ciprofloxacin	GCE + MB	SWV	<2 h		[142]
CTCs from Gallbladder Cancer	Gemcitabine and cisplatin	Tri-QDs/PEI@SiO2	SWASV		10 cells/mL	[143]
Colorectal tumor biopsy	Staurosporine, FOLFOX, FOLFIRI, cisplatin, YM-155 and MOC	EAB sensor using MB-Cyt C aptamers	SWV, KDM	100 s	1 ng/mL	[144]

Drugs: FOLFOX: fluorouracil and oxaliplatin; FOLFIRI: fluorouracil and irinotecan; YM-155: sepantronium bromide; MOC: mocetinostat. Electrodes: GCE: glassy carbon electrode; CPE: carbon paste-electrode; SPGE: screen-printed gold electrode; SPCE: screen-printed carbon electrode; SPSE: screen-printed silver electrode; GE: graphite electrode; ITO: indium-tin oxide glass substrate; AuME: gold microelectrode; GR/SPE: graphene screen-printed electrode; EAB: electrochemical aptamer-based. Materials: AuNPs/MWCNTs/SPCE: gold nanoparticles/multiwalled carbon nanotubes; Fe_3_O_4_@Au_T-PTA: gold-capped magnetic nanoparticles/synthetic temporin-PTA; CNT-SPE: carbon nanotube-SPE; SPGE-Van: Vancomycin; HRP-labeled dNTP/SPGEs: horseradish peroxidase-labeled dNTP/SPGEs; l-CeONP/ITO: L-lysine functionalized cerium oxide nanoparticle coated indium tin oxide; PEI-f-CNT: polyethylenimine-functionalized-carbon nanotube; PLL/MWCNT/GC: multi-walled carbon nanotube (MWCNT)-poly-L-lysine (PLL) hybrids; ECIS: electric cell–substrate impedance sensing; Ab/DNA/GO/GCE: DNA and monoclonal anti-bevacizumab antibodies/graphene oxide; E-Si-CRISPR: silver metallization into an electrochemical CRISPR-based biosensor; AmAuCPE: 3-APBA (3-Amino Phenyl Boronic Acid) modified AuCPE; Tri-QDs/PEI@SiO_2_: Trilayer-quantum dots/polyethylenimine (PEI)-encapsulated SiO_2_; SAM: self-assembled monolayer; mAb: monoclonal antibody; PBNC: prussian blue nanocubes; BL-Ab: β-lactamase monoclonal antibody; VAN/BSA: vancomycin chemically bound to bovine serum albumin; AuNPs/MXene + anti-PBP2a: composite made of anti-penicillin binding protein 2a bound to gold nanoparticles added to nanozyme; SPCE in PDMS ε-µD; PLL: screen-printed carbon electrodes in polydimethylsiloxane-based electrochemical microfluidic device with electrodes funtionalized using poly-L-lysine; CTX-AuSPEs: cefotaxime modified gold screen-printed electrodes; GCE + MB: glassy carbon electrode with methylene blue electron mediator; MB-Cyt C: methylene blue modified cytochrome c aptamers. Methods: CV: cyclic voltammetry; LSV: linear sweep voltammetry; DPV: differential pulse voltammetry; SWV: square wave voltammetry; EIS: electrochemical impedance spectroscopy; SWASV: square wave anodic stripping voltammetry; KDM: kinetic differential measurement.

## 5. Scanning Electrochemical Microscopy (SECM) Applied in Drug Resistance Studies

SECM has excelled as a high-performance and versatile electroanalytical technique with applications spanning research fields such as energy, corrosion, and biochemistry [145]. It is a scanning probe technique that employs an ultramicroelectrode (UME) biased at a specific potential to scan across a surface while recording an electrochemical current with high temporal and spatial resolution [146]. One of the main features of this electrochemical method is the fact that it not only reveals the topography of the studied sample, but also maps its electrochemical properties [146,147,148]. SECM has been applied to study biological systems by detecting trace levels of biomolecules and metabolites consumed or expelled by living entities, such as cells and tissues [39,149]. It also successfully monitored cell interactions with redox-active molecules to generate insight into disease diagnosis and progression [148]. Therefore, this advanced electroanalytical method has become one of the most promising electrochemical tools to investigate the underlying mechanisms of AMR and CMR in biological samples and identify biomarkers for related cellular characteristics.

### 5.1. AMR Detection in Biofilms

The investigation of AMR using SECM has been mostly focused on the study of biofilms, since such bacterial structures are one of the main causes of drug resistance in several infections [150,151]. A biofilm is an organized community of bacteria surrounded by a self-produced matrix consisting of exopolysaccharides (EPS), which promotes adhesion to inert or living surfaces, including medical devices, dental materials, and biological tissues [151]. Biofilms can greatly exacerbate AMR due to the horizontal transfer of antibiotic resistance genes throughout the colony since the EPS matrix enables cells to exist in close proximity [152]. As a matter of fact, the transfer of resistance genes has been reported to be 700 times more efficient within a biofilm than among freely moving bacteria [153]. Inside the biofilm, antibiotics must overcome high bacterial density and an increased number of resistant cells that efficiently communicate with each other through signaling molecules, which is known as quorum-sensing (QS) [151].

**Figure 3:**
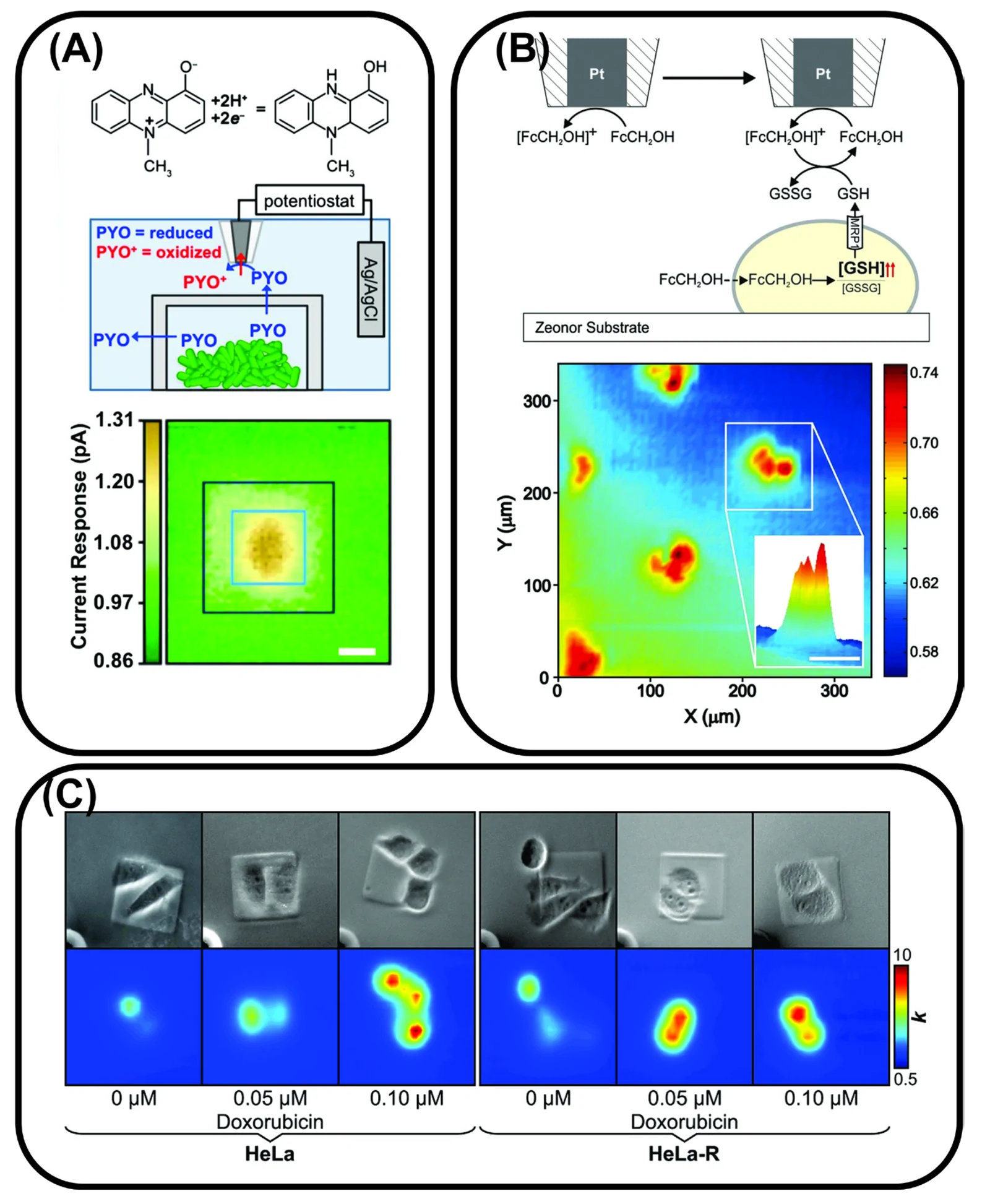
(**A**) Pyocyanin (PYO) redox reaction, a schematic of the microtrap-SECM system for measuring PYO in real time, and a SECM image for PYO collected above a microtrap containing *P. aeruginosa* cell in the study conducted by Connell et al. [155]. (Scale bar = 10 μm) (**B**) Schematic illustration of constant-height feedback mode SECM imaging with a living cell (HeLa) in the presence of FcCH_2_OH to investigate chemoresistance as reported by Kuss et al. [166]. FcCH_2_OH is oxidized to [FcCH_2_OH]^+^ at the Pt microelectrode and regenerated in proximity to the cell as a result of GSH efflux; a normalized SECM current image recorded at a distance of 12 μm above the substrate when HeLa cells were exposed to FcCH_2_OH (1 mM) for 75 min. (Scale bar = 50 μm) (**C**) Multidrug resistance-associated protein 1 (MRP1) functional activity of six (three populations for each cell line) different cell populations (wild-type HeLa cells and MRP1-overexpressing HeLa-R cells) obtained through a doxorubicin drug challenge. Optical micrographs (100 × 100 μm) of the cell populations during SECM imaging obtained by Polcari et al. [167].

QS is one of the main molecular mechanisms that regulate biofilm formation in bacterial colonies [150]. Due to the direct association of biofilm formation with increased drug resistance phenotypes, the investigation of QS can provide insight into the development of AMR. Given its high sensitivity and possibility to monitor biological systems with high resolution and in real time, SECM is an effective method that has been used to enhance the understanding of the redox chemistry of QS within a bacterial biofilm [154,155,156,157]. Connell et al. combined micro-3D printing techniques with SECM to probe QS-mediated communication in *P. aeruginosa* aggregates [155]. The micro-3D printing method was used to generate microtraps based on photo-cross-linked gelatin for bacterial growth. Using SECM imaging with a substrate generation/tip collection (SG/TC) operating mode, the authors monitored pyocyanin (PYO) as a redox-active biomarker of QS (Figure 3A). This molecule is a secondary metabolite involved in *P. aeruginosa* virulence, and whose biosynthesis is strictly controlled by QS. PYO can diffuse from the microtraps through the permeable gelatin polymer and reach the SECM platinum tip, where it is oxidized. This enabled the authors to quantify PYO production in the aggregates and determine the number of cells required for this bacterial species to initiate QS. It was demonstrated that QS can occur in bacterial aggregates as small as 500 cells, and that aggregates with a population higher than 2000 cells are required to stimulate QS in neighboring aggregates positioned 8 μm apart. This study introduced the combination of micro-3D printing with SECM as a powerful strategy to study the influence of spatial organization and aggregate population size on bacterial behavior in biofilms and could be expanded to investigate drug resistance phenotypes.

Besides QS biomarkers, SECM has been used as a valuable tool to study the redox chemistry of a bacterial biofilm. By deepening the understanding of the redox properties of such biological communities, insight into cellular physiology can be generated, which directly includes AMR in drug-resistant microorganisms. Klementiev et al. efficiently utilized SECM with an SG/TC operating mode to investigate a biofilm consisting of the oral pathogen *Aggregatibacter actinomycetemcomitans* and the oral commensal *Streptococcus gordonii* [156]. The developed setup consisted of a micromanipulator and a potentiostat, which allowed the precise positioning of 25-μm platinum UMEs over the scanned surface while accurately recording the electrochemical current. After artificially producing bacterial biofilms using noble agar on stainless-steel disc surfaces, the authors were able to quantify catalase-mediated H_2_O_2_ degradation by *A. actinomycetemcomitans* cells using electrochemical imaging. In addition, chronopotentiometry analysis was employed to map redox potentials over the investigated biofilms, which is related to bacterial metabolism and antibiotic efficacy [158,159]. It was demonstrated that the *S. gordonii* and *A. actinomycetemcomitans* biofilms present levels of oxidized species that are 2.4 times higher on average when compared to the *A. actinomycetemcomitans* biofilm. According to the authors, one of the factors responsible for more oxidized biofilms containing *S. gordonii* cells might be related to the large amounts of L-lactic acid produced by this bacterial species, which decreases the medium pH. This study demonstrates that scanning electrochemical microscopy is valuable to measure not only current profiles in biofilm samples, but also redox potentials with high spatial and temporal resolution.

SECM has been constantly evolving and combined with other analytical techniques to enable its application in the study of biological samples, including bacterial biofilms. For instance, SECM-atomic force microscopy (SECM-AFM), SECM-fluorescence microscopy, and scanning ion conductance microscopy (SICM) have been successfully employed to help elucidate biofilm dynamics and its redox chemistry [154]. Besides that, the use of alternative scanning probes, such as soft probes, has been recently reported to increase the method’s efficiency in biofilm electrochemical research. This aspect was explored in studies recently conducted by Darvishi et al. [157,160]. Soft-probe SECM was employed in feedback operating mode to study biofilms of *Escherichia coli* strains presenting susceptibility and resistance to the antibiotic ampicillin [160]. The soft probe consisted of flexible thin PET sheets containing a carbon paste microelectrode and was utilized to image the biofilms non-invasively in the presence of FcCH_2_OH as the redox mediator. A significant increase in FcCH_2_OH oxidation current compared to bare glass was observed when the soft probe was in contact with the biofilms, because of the FcCH_2_OH regeneration by the respiratory electron transport chain of living *E. coli* cells. Ampicillin-resistant cells generated higher electrochemical currents when compared to susceptible cells, which suggests their higher metabolic activity. Further treatment of the biofilms with the antibiotic gentamicin caused a drastic current decrease, which confirms the efficiency of the soft-probe-based SECM approach to study biofilm activity and differentiate drug resistance phenotypes.

Studies that employ in vivo models to monitor AMR and CMR using electrochemical sensors have been described in the literature. For example, Thirabowonkitphithan et al. modified SPCEs with a MWCNTs/PVA hydrogel and investigated sensing properties towards pyocyanin using SWV in various sample matrices [161]. In this study, the sensor was able to detect pyocyanin in burn wounds simultaneously infected with *P. aeruginosa*, *S. aureus,* and *E. coli* on porcine skin (ex vivo) and in living mice (in vivo). To this end, *P. aeruginosa* was inoculated into the center of the wound on the mouse skin and after 2 days of bacterial inoculation, the mouse was anesthetized, and the sensor was fixed to the wound. Subsequently, SWV analyses were successfully carried out to detect pyocyanin oxidation in the wounds, showing that the proposed sensor successfully identified *P. aeruginosa* infection and indicated the potential development of AMR.

Building on this work, Wang et al. advanced the field by introducing a novel, high-throughput platform to evaluate antimicrobial susceptibility of biofilms [162]. The group integrated a structured biofilm microarray with SECM and used resazurin as the redox mediator to monitor E. coli and P. aeruginosa biofilm metabolic activity in real time. They investigated the metabolic activity by comparing the reduction of resazurin by active intracellular reductases within the living biofilm to the reduction of resazurin at the 10 µm UME after it was biased at 0.6 V vs. Ag/AgCl reduction potential. Using the redox competition operating mode in SECM, as the UME scanned across the microarray spots, the researchers could measure the competition between the biofilm and the UME for the localized concentration profiles of resazurin and its reduced forms under varying antibiotic concentrations (ciprofloxacin and benzalkonium bromide) over time. Metabolically active biofilms reduce resazurin, resulting in less oxidized mediator at the UME surface, ultimately reducing the measured voltammetric current. Their findings successfully quantified dose-dependent and time-dependent decline in metabolic activity with increasing antibiotic concentrations without disrupting the physiological homeostasis of the microbial communities. This non-destructive and high-throughput platform is essential for rapidly identifying personalized antibiotic combinations efficiently, allowing clinicians to test multiple conditions simultaneously.

Preceding studies have established essential frameworks for mapping biofilm signaling and topography, but the recent work of Zhou et al. goes a step further to investigate how the physical stiffness of the 3D environment surrounding biofilms actively contributes to antibiotic resistance [163]. In this study, they developed an in vitro biofilm model using agarose gels with three varying stiffness (soft, moderate, and stiff) to mimic mucus and tissue of the human host. Then, programmable pulse potentials were applied to a 10 µm platinum UME to track local respiration and reactive oxygen species (ROS) signals in single embedded *E. coli* aggregates in situ during gentamicin exposure. Interestingly, competitive and SG/TC operating modes were employed for local respiration and ROS quantification, respectively. Real-time SECM data revealed that biofilms in stiffer gels have decreased respiration activity and display distinct ROS profiles when compared to softer gels. Subsequent RNA sequencing showed that stiffer gels also promote extracellular polymeric substance (EPS) production. Hence, both findings link altered metabolism and enhanced EPS production to increased antibiotic tolerance. Moreover, SECM data for oxygen reduction that was used to assess biofilm respiration supported the conclusion that combining glycine with gentamicin improves antibiotic efficacy. When SECM metabolic data was combined with the MBEC_50_ viability assay, it was revealed that the ∆*yjbE* mutant, which was a knockout of the upregulated EPS gene, had reduced survival in stiff agarose gels in the presence of gentamicin. Together, these findings demonstrate SECM as a promising tool to connect host tissue stiffness, metabolic activity, and EPS production in biofilms and how they contribute to antibiotic tolerance. The findings also explain why antibiotics that appear to be effective in 2D assays can fail against biofilms embedded in dense tissues, like in chronic wound infections caused by *P. aeruginosa*.

### 5.2. CMR Detection

The study of CMR by SECM has been mostly focused on the presence of multidrug resistance efflux pumps in the plasma membrane of tumor cells. Such protein pumps play a crucial role in the development of chemoresistance in cancer cells, being one of the most common mechanisms of resistance to drugs [164], which ultimately leads to reduced treatment effectiveness and drastically reduces patient survival rates. P-glycoprotein (Pgp) and multidrug resistance protein 1 (MRP1) pumps are known to be overexpressed in cancer cells that acquire resistance, acting as authentic molecular pumps that actively remove drug molecules from inside the cells, preventing them from exerting their therapeutic effect [164].

In SECM research, most of the studies on chemoresistance in cancer cells have been centered on the MRP1 efflux pump [40,165,166,167]. In their study published in 2011, Kuss et al. [168] compared the electrochemical current profile of wild-type human adenocarcinoma cervical cancer cells (HeLa) with a multidrug-resistant variant known to overexpress MRP1 (HeLa-R). Both cell lines were imaged using SECM in constant-height feedback mode in the presence of ferrocenemethanol (FcCH_2_OH) as a redox mediator. Employing flow cytometry experiments, the authors observed that the presence of FcCH_2_OH induced an increase in the intracellular levels of glutathione (GSH). Its excess is readily expelled from the cells through MRP1. In the extracellular medium, GSH reacts with ferrocenium methanol ([FcCH_2_OH]^+^) and reduces it back to FcCH_2_OH. This constant regeneration is responsible for the increase in the electrochemical current recorded in SECM measurements whenever the platinum UME scans over the cancer cells. A differential electrochemical current profile was observed when comparing HeLa and HeLa-R cells, with the resistant variant originating a lower current response. The regeneration mechanism of FcCH_2_OH mediated by GSH reported in this research represented an important achievement for the study of chemoresistance in cancer cells using electrochemistry. Through these findings, we (under the lead of J. Mauzeroll) consistently proved that the redox state of cells and their drug-resistant phenotypes can be successfully measured using SECM.

As a continuation of this study, our research efforts [166] reported the extraction of kinetic information on the regeneration of FcCH_2_OH by wild-type HeLa and resistant HeLa-R cancer cells in SECM measurements (Figure 3B). The ability of cells to regenerate the redox mediator was quantified and expressed as an apparent heterogeneous rate constant ($k_{0}$), which could serve as a numerical indicator of chemoresistance. Cells were patterned and imaged electrochemically using cell-permeable (FcCH_2_OH) and impermeable ([Ru(NH_3_)_6_]^3+^) redox mediators, which allowed the authors to separate topography and reactivity contributions to the cell’s electrochemical current response. The $k_{0}$ values obtained after mathematical treatments proved to be significantly different when comparing HeLa and HeLa-R cells, with the latter displaying a higher electrochemical activity and faster kinetics when compared with the non-resistant cell line. The successful decoupling of topography from cell reactivity, and the estimation of the apparent heterogeneous rate constant, made a significant contribution towards the quantification of chemoresistance in human cancer using SECM.

The studies by Kuss et al. [166,169] were used as a foundation for the research conducted by Polcari et al. [167], in which the functional activity of MRP1 efflux pumps was directly measured by SECM with feedback mode in single HeLa and HeLa-R cells in the presence of the chemotherapeutic drug doxorubicin. Both cell lines were exposed to different concentrations of the drug to evaluate potential differences in the expression and activity of MRP1 pumps. SECM analysis in the presence of FcCH_2_OH and [Ru(NH_3_)_6_]^3+^ mediators enabled the extraction of the *k*_0_ for each cell type representing MRP1 activity, whereas flow cytometry experiments revealed the cellular expression levels of MRP1 pumps after exposure to the drug. The authors found out that the treatment of cells with doxorubicin resulted in increases in both the expression and activity levels of MRP1 for non-resistant and resistant HeLa cells. However, functional activity and expression did not directly correlate, as the MRP1 activity measured in wild-type HeLa cells did not significantly differ from resistant HeLa-R. These findings were relevant because they suggest that the measurement of the functional activity of MRP1 should be prioritized over expression for a better understanding of chemoresistance in cancer cells.

Kuermanbayi et al. further built on this work by investigating the influence of the extracellular matrix stiffness on MRP1 efflux pump activity of two different breast cancer cell lines (MCF-7 and MDA-MB-231) (Figure 3C) [40]. Considering that the extracellular matrix stiffness is known to affect the resistant behavior of cancer cells towards certain chemotherapeutics [170], the authors cultured the two cell lines using polyacrylamide gels with three different degrees of stiffness to mimic benign, malignant, and advanced stages of breast cancer. SECM was then employed using feedback mode to investigate the functional activity of MRP1 in the cell lines studied before and after treatment with vincristine (a chemotherapeutic known to be expelled from cancer cells through MRP1), using FcCH_2_OH-GSH interaction as an electrochemical indicator. In a similar way to the studies described previously, the efflux activity of MRP1 was quantified by extracting a regeneration rate (*k*) for FcCH_2_OH by the cells. It was found out that the increase in the extracellular matrix stiffness induced an increase in the MRP1 efflux activity towards vincristine, with the MCF-7 cells showing a more pronounced increase than the MDA-MB-231 cells. Also, it was demonstrated that the MRP1 activity in MCF-7 cells was more significantly influenced by extracellular matrix stiffness than that of MDA-MB-231 cells. This study was the first to employ SECM to quantitatively investigate the influence of different tumor mechanical micro-environments on the resistant phenotypes of cancer cells.

More recently, our group has also shown the first effective application of SECM in feedback mode to detect carboplatin (CBDCA) chemoresistance in living epithelial ovarian cancer cells [171]. Using FcCH_2_OH as a redox mediator to quantify the efflux of glutathione (GSH), a key biomarker for cellular drug resistance, Daou et al. [171] compared the electrochemical responses of drug-susceptible A2780-S and platinum-resistant A2780-CP cell lines. Initially, both cell types exhibited similar metabolic redox activity and GSH levels in the absence of the drug. However, upon exposure to 20 mM CBDCA, susceptible A2780-S cells showed a significant decrease in electrochemical current, indicating an inability to cope with drug-induced oxidative stress. In contrast, resistant A2780-CP cells displayed a distinct increase in current that stabilized over time, suggesting they successfully mitigate cytotoxic effects. Using numerical modeling previously established by our group and collaborators [38,169,172] to decouple topography from reactivity, the study extracted apparent heterogeneous rate constants ($k_{0}$) which confirmed that resistant cells decrease GSH efflux to cope with the drug, likely through intracellular enzyme-catalyzed drug detoxification or reduced membrane permeability.

This research highlights SECM as a rapid and highly sensitive tool capable of distinguishing between resistant and susceptible cancer cells within minutes. Based on the examples discussed, trends between SECM operating modes and the resistance mechanism under investigation can be identified. In the case of cells or biofilms that consume a molecule that is itself electrochemically active, competition modes are attractive. This is true, for instance, with mechanisms involving the consumption of O_2_, such as the work of Zhou et al. [163]. Alternatively, the starting molecule may be difficult to reduce or oxidize at the electrode; however, if products generated by a biological mechanism are electroactive, then SG/TC mode is useful. This can be observed in the investigation by Klementiev and Whiteley [156]. Lastly, systems that involve the use of a redox mediator that interacts with and is regenerated by a biochemical pathway associated with AMR or CMR can be easily studied in feedback mode. This is exemplified by the examples by Darvishi, Kuss, and Polcari [157,160,166,167,168,171].

## 6. Impact Electrochemistry

One of the most promising advances to date is impact electrochemistry, a powerful tool for the detection of single redox-active entities based on their collision with an electrode [173,174]. Briefly, its principle relies on the faradaic charge transfer of suspended single particles upon their collision with an electrode that is biased at a constant potential, usually exceeding the standard redox potential of the detected species or material. Therefore, current bursts (spikes) are recorded in the chronoamperometric curve for each collision event taking place at the electrode surface [175,176]. The effectiveness of this electrochemical approach was already proven with metallic and metal oxide nanoparticles [177,178,179], biomolecules [173,174,180], and, more aligned with the scope of the present review, living bacteria [176,181] and cancer cells [182].

In an interesting study, Couto et al. detected *E. coli* cells via impact electrochemistry employing *N*,*N*,*N’*,*N’*-tetramethyl-*para*-phenylene-diamine (TMPD) as a redox mediator to enable the electrochemical recognition of bacterial cytochrome *c* oxidases [176]. As *E. coli* cells present cytochrome *c* oxidase in their plasma membranes, they can enzymatically oxidize extracellular TMPD to TMPD^•+^ radicals. A carbon microelectrode was biased at a cathodic potential to drive the reduction of the radical cation TMDP^•+^ present in solution back to TMPD. When the bacteria enter the diffusion layer, a feedback redox cycle is thus created, and an increase in cathodic current is detected during chronoamperometry measurements, which corresponded to the regeneration of TMPD^•+^ by bacterial cytochrome *c* oxidase [176]. On the other hand, Dick et al. employed impact electrochemistry to differentiate healthy thymocytes from acute lymphoblastic lymphoma T-cells (T-ALL) in the presence of the surfactant TritonX-100 [182]. The surfactant increased cell permeability and enabled electroactive cellular compounds to be detected electrochemically whenever a cell collided with a gold microelectrode biased at −1.0 V. Interestingly, T-ALL cells generated current collision peaks that differed from the studied healthy cells by two orders of magnitude, which was ascribed to the different content of redox-active metabolites in the cancerous cells. These two studies exemplify the valuable contributions of impact electrochemistry that could be incorporated in the design of new strategies to investigate and detect AMR and CMR.

To our knowledge, no in-depth AMR or CMR impact electrochemistry study in complex media exists. However, studies that detect small molecules and genetic material in complex media establish a promising precedent for this work. Gu et al. demonstrate an interesting intercellular nanoelectrode for impact electrochemistry inside living U2OS cells [183]. Treatment with arsenite stimulated cellular formation of stress granules containing reactive oxygen and nitrogen species (ROS and RNS). Upon contact between the granules and the nanoelectrode, to which a potential of +800 mV vs. Ag/AgCl was applied, various ROS and RNS species could be detected. It should be noted that while the authors state minimal interference in the intercellular environment, more severe interference and lower signal-to-noise ratios would likely be encountered in clinical samples such as blood or urine. Interestingly, they report a gradual decrease in collisions over time, which they attribute to electrode fouling. Yuan et al. report the detection of miRNA using ssDNA functionalized silver nanoparticles (AgNP) using impact electrochemistry in human serum samples [184]. Upon binding of miRNA to the ssDNA-AgNP, enzymatic cleavage of the resulting double-stranded DNA is accomplished by the addition of DSN, thereby exposing the metallic surface of the nanoparticle, enabling its electrochemical oxidation. This was achieved in authentic human serum with an LOD of 0.011 fm. While these two examples do not explicitly demonstrate AMR- or CMR-based detection using impact electrochemistry in complex media, they highlight the detection of small molecules and genetic materials related to AMR or CMR in such media.

While these examples demonstrate the potential for impact electrochemistry for quantifying resistance, there are still substantial barriers that must be addressed. Key issues include electroactive components of clinical samples that can cause substantial background noise collisions or fouling of electrode surfaces. While strategies such as employing highly specific enzymatic nanoparticle-based assays have been effective, the commercialization of such approaches may prove challenging. While scalable commercial manufacturing of ssDNA and AgNP is available, concerns regarding their shelf-life and strict reproducibility remain. Research on impact electrochemistry for the detection of AMR or CMR in complex media is therefore required, particularly with commercially viable approaches and antifouling electrode coatings.

When comparing the two emerging electrochemical techniques in this field, notably SECM and impact electrochemistry, one can arrive at several important contrasts. The sample throughput for impact electrochemistry can be significantly faster than SECM. The latter technique often involves cells being extracted from samples, cultured, and then plated prior to even beginning analysis. On the other hand, impact electrochemistry can often be performed directly on a sample; however, some preparation or separation steps may be required to remove interferants. However, SECM offers superior spatial resolution on the micro or even nano scale depending on the geometry of the electrode. While impact electrochemistry can utilize small nanoelectrodes, as seen with Gu et al., it does not offer spatial resolution for local electrochemical activity. While both systems require a trained electrochemist to operate, SECM is arguably a more complex technique for AMR and CMR investigations. This is especially true as the effect of experimental parameters, such as media composition, light exposure, and temperature, on the electrochemical signal must be well-understood [147,185].

## 7. Discussion and Outlook

### 7.1. Problems and Threats Caused by AMR and CMR

Antimicrobial resistance (AMR) and chemoresistance (CMR) pose significant threats to global public health. Perhaps the most notable manifestations of drug resistance are increases in mortality and morbidity. AMR leads to the failure of standard antibiotic treatment, making common infections more difficult to treat and sometimes even incurable. This has led to long-term illness, higher mortality rates, and an increased risk of infection complications that were once easily controlled. The infections caused by multidrug-resistant ‘superbugs’ are associated with high mortality rates, such as cases of methicillin-resistant Staphylococcus aureus (MRSA) [133] and carbapenem-resistant Enterobacteriaceae (CRE) [186]. Similarly, CMR leads to a decrease in the effectiveness of chemotherapy drugs, allowing tumors to continue growing and metastasizing, worsening the patient’s condition. Drug-resistant cancer patients are also more likely to relapse after treatment. This often leads to more invasive and difficult-to-treat cancers in the future. Another symptom of drug resistance is the strain upon the medical system. Infections caused by drug-resistant bacteria typically require longer hospital stays, and patients may need to be isolated to prevent the spread of drug-resistant bacteria, which further increases treatment costs. When first-line antibiotics fail, healthcare providers must turn to more expensive and potentially more toxic alternatives. These treatments typically require deeper monitoring and supportive care, increasing the overall cost of healthcare [187]. Cancer chemotherapy-resistant tumor patients typically require complex treatment plans, including combinations of different drugs, higher doses, or experimental therapies. These treatments are more expensive and may not be covered by insurance, imposing an economic burden on patients and the healthcare system [188].

AMR and CMR also result in significant economic implications as the cost of treating drug-resistant infections is significantly higher than that of treating non-drug-resistant infections. It is estimated that if current trends continue, antibiotic resistance could lead to cumulative economic losses of up to $100 trillion by 2050 [7], with low-income countries bearing the brunt. Treating chemotherapy-resistant cancer requires more resources, resulting in higher direct medical costs. This includes the cost of new drugs, additional testing, and longer treatment times. The economic impact of CMR is not limited to healthcare costs but also includes productivity losses and the economic burden on families with long-term illnesses. The economic chain reaction can be far-reaching, especially in a society where medical expenses are borne by patients at their own expense. With the continuous rise in AMR, people are increasingly concerned about the ethical impact of limited opportunities for effective treatment. This is particularly true in low- and middle-income countries, where the cost of effective new antibiotics may be too high. The use of experimental or unproven therapies to treat chemotherapy-resistant cancer has raised ethical concerns about informed consent, particularly in cases where patients urgently need treatment options [189]. Patients with chemotherapy-resistant cancer often face significant psychological and social challenges, including anxiety, depression, and economic pressure. These factors can lower the quality of life and complicate treatment compliance.

### 7.2. Advantages, Challenges, and Current State of Electrochemical Methods

Electrochemical approaches have been identified as promising methods to monitor AMR and CMR in bacterial infections and cancer progression due to their high sensitivity, fast response, and portability. The utilization of such methods to create new drug susceptibility assays will enable healthcare professionals to rapidly develop personalized treatment regimens, thereby avoiding empirical treatments and the unnecessary exposition of patients to different medications and doses.

Electroanalytical techniques, such as voltammetry and electrochemical impedance spectroscopy (EIS), are highly sensitive, capable of detecting very low concentrations of target molecules, including biomarkers of AMR and CMR [35]. The specificity of these methods can be enhanced by modifying electrodes with nanomaterials, biomolecules, or specific polymers, allowing for the targeted detection of specific drugs or resistance-related biomarkers. When considering response time, electrochemical assays can deliver results in minutes or even seconds; this rapid turnaround is crucial for timely decision-making in clinical settings. Furthermore, they are capable of real-time monitoring of biological processes, making them ideal for applications requiring dynamic analysis, such as tracking the efficacy of a therapeutic intervention. Lastly, electrochemical sensors can be miniaturized into portable formats, such as point-of-care (POC) devices, including remote or resource-limited areas. Integration of these small architectures with wearable devices for continuous monitoring of drug levels or biomarkers in real-time provides ongoing data without the need for invasive procedures [18,190].

This brings us back to the initial question about whether it is genomic or cell activity-based methods are more promising for the detection of drug susceptibility and resistance in laboratory and clinical samples. While genomic assays can be incredibly accurate and have extremely low detection limits, they are restricted by our knowledge of genetic and biological markers correlating to drug resistance. In other words, if we do not know if a certain gene correlates to drug resistance, it cannot be quantified. On the other hand, activity-based techniques are far more flexible in the sense that even contributions toward drug resistance that are not fully understood are still taken into consideration when assigning a resistance phenotype. This is the case for most clinical samples. Activity-based techniques are often more sensitive to matrix effects; therefore, extra precautions must be taken to limit this interference.

Electrochemical assays generally involve lower costs per test, making them more accessible, especially in low-resource settings. This is especially true for many of the activity-based assays, which utilize unmodified or lightly modified electrodes, as they can easily be scaled down onto inexpensive screen-printed electrodes for small disposable test kits. Depending on the design, electrodes used in electrochemical sensors can also be reusable, further reducing costs associated with diagnostics. For example, consider the AMR proof-of-concept developed by our group [27], which can be integrated into a reusable microfluidic platform for a cost-effective, portable, and reusable device. Electrochemical methods can be applied to a wide variety of samples, including blood, urine, and environmental samples, making them versatile tools for clinical diagnostics, environmental monitoring, and further reducing costs by means of sample preparation and extraction. This robust handling of matrices has been demonstrated by several of the presented examples, such as the ECIS sensor developed by Ralston et al. [123], which successfully analyzed untreated urine samples for bacteria associated with UTIs, notably with zero false negatives. The same electrochemical platform can often be adapted to detect different analytes by simply changing the biorecognition element, such as antibodies, aptamers, or enzymes [191].

While electrochemical methods can be miniaturized, integrating all the necessary components (sensors, power supply, data transmission, etc.) into a single portable device is complex and requires interdisciplinary collaboration. Moving from laboratory-scale research to commercial production requires significant industrial support, which has been limited. Developing scalable manufacturing processes that maintain sensor performance is also a significant challenge. Effective development of electrochemical sensors for clinical use requires collaboration between chemists, biologists, engineers, and clinicians. The lack of such interdisciplinary collaboration has hindered progress in this field.

There are several challenges facing the widespread commercialization of biosensors. In fact, the first commercial blood glucose sensor was developed 50 years ago, but it still dominates 56–85% of the biosensor market [192,193]. While this can be attributed to many factors, including the massive market of diabetes management, it clearly illustrates a lack of biosensor commercialization for other applications in the field.

While many of the proof-of-concept studies published in recent years boast impressive performance in the laboratory, they utilize sophisticated and complex mechanisms for signal transduction. Such designs are vulnerable to fouling, contamination, interference, and other negative effects encountered in heterogeneous encountered in clinical sensing. This is one of the major factors impeding FDA/EMA approval for novel biosensors. Chemical separations and purifications could be applied to remove components of the heterogeneous media; however, this would increase the complexity and cost of these biosensor-based assays. One interesting solution that has been suggested to overcome this issue is to acquire large datasets and apply AI or machine learning for calibration to overcome challenges associated with the heterogeneous nature of these samples [194]. While possible solutions exist, prioritizing robust sensor designs which are less susceptible to these factors should take precedence. This is exemplified by the antifouling strategies discussed earlier in the text.

Another pragmatic aspect that is often overlooked when developing a sensor is the economic context of the final device. More specifically, the possible customer base, user needs, cost of materials, supply chain considerations, and manufacturing scalability/reproducibility. For instance, academic facilities often possess high-precision fabrication instruments and highly trained personnel; this may not be readily available for early commercialization stages. However, new technologies such as 3D printing and microfluidics offer alternatives to more advanced nano- and microfabrication techniques that are more accessible and economically viable for the production of biosensors. Researchers frequently neglect to carefully consider these important factors that can limit, or even prohibit, commercialization of biosensors. Often, the main considerations are analytical metrics such as response time, accuracy, and precision. A holistic approach predicated on the entire economic context of the final device, as described above, should be more carefully developed prior to initiating biosensor research projects. We direct the reader towards useful reviews which cover the topic of biosensor commercialization in-depth for additional details [193,195].

Aside from commercialization, more general technical challenges must be thoroughly addressed to develop practical biosensors. This includes electrode fouling, variations in clinical media composition, and reproducibility of biosensor components. Electrode fouling is one of the most significant obstacles, typically resulting in signal drift or gradual intensity decreases, as seen with Gu et al. [183]. Such observations may be erroneously mistaken for drug susceptibility. Electrode coatings discussed in Section 3 offer possible solutions to fouling; however, their commercial viability and effect on sensor performance must be carefully considered and evaluated. Variations in the composition of clinical media are another hurdle for practical application of electrochemical biosensors. For example, consider blood samples from a variety of patients. Depending on the composition, cell counts, and hydration of the individual patient, the viscosity and, consequently, the mass transport properties will vary. Ultimately, this will affect diffusion of analytes to the electrode and can alter electrochemical signals, leading to false positives or negatives for AMR/CMR determinations. Reproducibility of biosensor components and signals is also a major concern. This is particularly true for biosensors which utilize nanomaterials or other highly sophisticated mechanisms and materials. For example, some polymeric coatings or hydrogel layers may have poorly defined chemical structures with varying individual polymer lengths or cross-linking. Research is therefore required to address these technical challenges.

### 7.3. The Role of SECM in Advancing Drug Resistance Research

SECM is a powerful technique that has significantly advanced drug resistance research, particularly in understanding AMR and CMR. SECM’s ability to provide real-time insights, with high spatial and temporal resolution, into cellular and molecular processes has made it a critical tool for studying how drug resistance develops and functions in living entities. Specifically, the studies discussed in this review are successful examples of the efficiency of SECM to measure the redox state of cells and detect drug-resistance phenotypes, both in cancer and bacterial biofilms.

One of the primary mechanisms behind chemoresistance in cancer is the overexpression of drug efflux pumps such as P-glycoprotein (Pgp) and Multidrug Resistance Protein 1 (MRP1). SECM has been used to map efflux pump activity in real time, quantifying how drugs like doxorubicin or cisplatin are transported out of cancer cells. This allowed researchers to understand how different cancer cells express and use efflux pumps to resist chemotherapy, which can guide the development of more effective treatments. Furthermore, SECM provides the ability to analyze drug efflux activity at the single-cell level, allowing researchers to distinguish between drug-resistant and drug-sensitive cells within a heterogeneous tumor population. This is critical for understanding how small subpopulations of cancer cells survive chemotherapy and later cause relapses. SECM can quantify the kinetics of this drug transport, providing data on how fast and how efficiently cancer cells expel chemotherapeutic agents. These kinetic insights can be used to develop efflux pump inhibitors, which block the resistance mechanism and enhance the efficacy of chemotherapy [149].

Electrochemical activity of three-dimensional biofilms can also be investigated using SECM to elucidate key details regarding drug resistance and the underlying mechanisms. This helps in identifying which parts of the biofilm are most resistant to antibiotics, allowing for targeted interventions. SECM can detect redox-active quorum-sensing molecules, such as pyocyanin produced by *P. aeruginosa* [155]. By monitoring QS molecules in real-time, SECM helps researchers understand how bacterial communities coordinate resistance within biofilms, offering potential strategies to disrupt these communication pathways [151].

In both AMR and CMR, drug resistance is often associated with metabolic changes in cells. SECM is highly sensitive to redox changes, which makes it an excellent tool for monitoring these metabolic shifts. SECM can detect changes in the redox state of cells as they develop resistance. For example, cancer cells may alter their glucose metabolism or increase antioxidant production to protect against chemotherapy. SECM can track these metabolic shifts and identify biomarkers of resistance, which could be targeted in new therapeutic strategies. Efflux activity of resistant cells can be detected by detecting changes in redox mediators, such as glutathione (GSH) [40]. This is critical for understanding how cells detoxify chemotherapeutics or antibiotics by pumping out drugs or altering their redox chemistry.

Drug resistance is often a dynamic process, with cells acquiring resistance over time through genetic mutations or adaptive responses. SECM’s ability to perform continuous real-time measurements allows researchers to monitor efflux with excellent temporal resolution. This is achieved by monitoring drug-resistant cells or bacteria as they are exposed to drugs, tracking how their redox states or efflux activity change over time. Such studies have helped researchers understand the time dynamics of resistance development, which is essential for designing drug regimens that prevent resistance from emerging. This is advantageous compared to traditional methods, as SECM allows for in situ detection of resistance markers in living cells or bacteria without the need for complex sample preparation [149].

It is also intriguing to note that SECM is increasingly being integrated into drug discovery pipelines to help identify compounds that can overcome AMR and CMR. SECM can be used to screen potential efflux pump inhibitors, which are compounds designed to block the action of drug-resistance pumps. By monitoring how cells respond to these inhibitors in real-time, SECM provides data on their efficacy and helps optimize their design. SECM can be applied in combinatorial therapy testing, where it is used to observe how different drug combinations affect resistant cells or bacteria. This allows researchers to identify synergistic drug pairs that could bypass resistance mechanisms or enhance drug penetration in biofilms and tumors [196]. In fact, recent work from our lab highlights SECM as an essential tool for identifying new avenues for drug discovery [171]. Specifically, it allows researchers to evaluate the efficacy of drugs targeting cell permeability or those inhibiting intracellular drug detoxification enzymes, such as glutathione-S-transferase, to combat chemoresistance.

The continued development of SECM is expected to play a pivotal role in personalized medicine and precision oncology. Particularly promising is the application to generate personalized drug resistance profiles for cancer patients by analyzing cells from biopsies or blood samples. This would allow clinicians to tailor chemotherapy regimens based on the specific resistance mechanisms present in the patient’s cancer.

### 7.4. Future Directions

Given the enormous promise of electrochemical methods for investigating AMR and CMR, and highly compelling proof-of-concept studies, it is important to suggest future strategies in this burgeoning field. While genomic and activity-based assays are both useful, the majority of the improvements and future research objectives we can identify are applicable primarily to activity-based assays. Although many experiments already utilize modified electrode surfaces, the further incorporation of nanomaterials, biomaterials, polymeric, or self-assembled monolayers can further improve the performance of electrochemical biosensors. This is especially important for SECM experiments wherein nearly all experiments utilize unmodified or lightly modified UME probes. It is also key for advancing activity-based assays to improve their function in untreated matrices common in patient-derived samples. Development of versatile high-performance probes featuring nanomaterials to increase sensitivity and spatial resolution or hydrogels that enable analysis of biofilms is urgently needed. SECM could be further developed to perform real-time monitoring of cellular changes during drug treatments, observing how resistance mechanisms are activated at different time points. This dynamic monitoring could lead to more effective and timely interventions, adjusting treatments based on real-time cellular responses. Once again, this is also true for activity-based assays, since they can actively monitor rapid changes in redox processes or impedance, whereas many biorecognition mechanisms utilized in genomic assays require binding of biomolecules with sluggish kinetics that would struggle to accurately represent real-time changes in cellular behavior.

Electrochemical techniques such as SECM can also play a key role in developing treatments for diseases. By studying the interaction between drugs and cells in real time, SECM could help identify how resistance evolves, guiding the design of next-generation drugs that can bypass or block these mechanisms. SECM could be used in high-throughput screening (HTS) platforms for discovering new antibiotics or chemotherapeutics [197]. Researchers could quickly test how different compounds affect the redox state or metabolic activity of resistant bacteria and cancer cells, enabling the identification of drugs that can overcome resistance mechanisms. SECM could also help identify synergistic drug combinations that target multiple resistance pathways simultaneously. For example, SECM could monitor how drugs affect both efflux pump activity and redox balance in cancer cells, helping researchers find the most effective combinations.

Future electrochemical technologies could be incorporated into wearable sensors that provide continuous monitoring of a patient’s infection or cancer progression. These sensors could track resistance biomarkers, such as bacterial metabolites or cancer cell efflux pump activity, in real time. This would allow for timely intervention, reducing the risk of treatment failure. Such devices could allow patients to be monitored at home, reducing hospital visits while enabling early detection of resistance. For cancer patients, this could minimize the side effects of unnecessary chemotherapy and allow physicians to adjust doses remotely based on SECM feedback.

In addition to its clinical application, the development of this method still has usage scenarios and potential in other areas in the future. SECM could be deployed in environmental monitoring systems to detect antibiotic residues in water or soil, helping to control the spread of AMR. This could be critical in areas near pharmaceutical factories, livestock farms, or hospitals, where antibiotic runoff is a significant concern. Electrochemical sensors could track the presence of AMR genes in environmental samples, allowing for real-time monitoring of how resistance is spreading in natural ecosystems. This could inform strategies to limit the environmental impact of antibiotics and prevent the emergence of new resistant strains [198,199].

Electrochemical sensors could also be integrated into livestock monitoring systems to detect early signs of infection and resistance in animals. This would enable farmers to make informed decisions about antibiotic use, reducing the overuse of antibiotics and limiting the development of resistance [200]. Similar devices could be used for real-time monitoring of soil health and microbial resistance in agricultural environments, which could help prevent the spread of resistance in plant pathogens and ensure that crops remain healthy without the overuse of agricultural antibiotics. It should be noted that activity-based assays would be more flexible across a variety of different agricultural and livestock species, given, for example, by detecting general indicators of oxidative cellular stress. Whereas for genomic analysis, the resistance genes for different organisms may differ significantly and require different biorecognition elements depending on the sensor.

## 8. Conclusions

Electrochemical approaches demonstrate an exciting potential to be widely applicable to the detection of drug resistance and research of the underlying mechanisms. Voltametric techniques and SECM enable the characterization of redox properties of drugs, including antibiotics and chemotherapeutics. Electrochemical methods have also contributed to the discovery of drug resistance biomarkers, such as glutathione. Proof-of-concept studies have shown enormous promise in applying these findings to detect and study both AMR and CMR. Due to their cost-efficiency compared to conventional techniques, the relatively simple operation of instrumentation, and fast acquisition time, electrochemical methods overcome many challenges of conventional resistance assays. In summary, considering the achievements of electrochemistry as a tool for the detection of AMR and CMR, it has the potential to greatly enhance traditional methods for diagnostics and disease treatment. The need for systems to detect drug susceptibility and resistance rapidly will ultimately demand the design and creation of POC devices that are portable and can be used by healthcare providers without the need for extensive training. The collaboration between academia and industry that focuses on the validation and product design of such POC devices will be crucial to make drug susceptibility testing a reality. It is our opinion that while genomic sensors can achieve impressive accuracy and low limits of detection, activity-based assays tend to be more flexible in their design, applicable to different systems, and more suitable for clinical drug resistance screening applications where it is possible not all genetic and/or biological markers are clearly understood (Scheme 1).

## Data Availability

No primary research results, software, or code have been included, and no new data were generated or analyzed as part of this review.

## Supplementary Materials

The following supporting information can be downloaded at: https://www.mdpi.com/article/10.3390/molecules31193566/s1 Table S1: Recent reports on electrochemical sensors to detect antibiotics and anticancer drugs.
