# Supplementary material for: Taking Charge Against the Silent Pandemic—Electrochemical Characterization of Drug Resistance
Source: Molecules. 2026 Oct 7;31(19):3566. doi: 10.3390/molecules31193566 (PMC13666848; doi:10.3390/molecules31193566)
Supplement: Supplementary file 1 [file molecules-31-03566-s001.zip › molecules-4471436-supplementary.pdf]

ELECTRONIC SUPPLEMENTARY INFORMATION

The charge against the silent pandemic –

Electrochemical characterization of drug resistance

Fangqing Liu[1], Michael A. Saley[1], Ana Carolina Mendes Hacke, Shubhneet Thind, Pauline Anne Barraquiel, Sabine Kuss*

Corresponding author: Sabine.Kuss@umanitoba.ca

[1] These authors contributed equally to this manuscript and are listed in alphabetical order.

Department of Chemistry, University of Manitoba, MB, Canada

Table S1 – Recent reports on electrochemical sensors to detect antibiotics and anticancer drugs.

Synthesized nanomaterial/ nanocomposite	Drug	Working electrode	Sample	Detection/ characterization technique(s)	Linear range	Limit of detection (LOD)	Reference
SnO_2/Bi_2S_3	Tobramycin	ITO	milk	EIS	5 - 50 nM	4.28 nM	45
AuNPs/RGO	Streptomycin	pencil lead graphite working electrode	milk	CV, EIS	10^{-8} - 10^{-16} M	0.8 x 10^{-18} M	46
SDS·Gr	Ciprofloxacin	CPE	urine samples	DPV	0.3 × 10^{-6} M - 100.0 × 10^{-6} M	0.03 µM	47
r-GOAC<br>r-GODC	Ciprofloxacin	CPE	fish tissues, urine and drug samples	CV, DPV	0.1–50.0 M<br>0.3–50.0 M	4.08 × 10^{-9} M 17.74 × 10^{-9} M	48
Mn_3O_4/g-C_3N_4	Sulfathiazole<br>Sulfapyridine	CPE	milk, honey and water samples	DPV	1–100 µM<br>0.75–100 µM	0.23 µM<br>0.11 µM	28
Gr-PLA	Tetracycline	3D-printed Gr-PLA	drug, tap water, and milk	BIA-AD	0.5–50 µM	0.19 µM	50
$[N(CH_3)_4]_2CoCl_2Br_2$	Sulfamethoxazole	ITO	-	EIS	10^{-11}–10^{-5} M	6.3 × 10^{-13} M	51
KAP/CMWCNTs/CNFs	Tetracycline	GCE	milk	DPV	1.0 × 10^{-17}– 1.0 × 10^{-5} M	2.28 × 10^{-18} M	52
N/Co@CNTs@CC-II	Nitrofurantoin	CC	milk and water samples	CV, LSV	0.05–550 µM	18.41 nM	53
Ni/MoN/MC	Metronidazole<br>Tinidazole<br>Ornidazole	GCE	tap water and tablets	DPV	5-20; 20-240 µM,<br>5-15; 15-150 µM 5-20;<br>20-300 µM	35 nM<br>8 nM<br>21 nM	49
MIP/Ag/Ni-MOF/N-GQDs	Olaquindox	GCE	fish muscle, swine muscle and chicken breast	DPV	5–600 nM	2.2 nM	54
GAgNPs	Ornidazole	CPE	milk	SWV	1.0 × 10^{-3} - 5.0 × 10^{-5} M	3.8 × 10^{-6} M	34
PANI-AgBr	Amoxicillin	SPCE	tap water<br>lake water	Chronoamperometry	1 – 500 ppb<br>1 – 500 ppb	0.728 nM<br>0.193 nM	55
Ag@POM@rGO	Ciprofloxacin	CPE	human plasma	SWV	0.103–122.88	0.031 µM	56
CNF	Amoxicillin	GCE	pharmaceutical	SWV	0.6 -3.0 µM	98 nM<br>95 nM	57

	Clavunate potassium		formulation and human serum samples				
Co-TCA@ZIF-67	Furaltadone	CPE	aquaculture water sample	DPV	50 nM - 5 µM	12 nM	58
CuMOF	Ciprofloxacin	GCE	tap water and sea water	DPV	2.5 – 100 µM	3.29 nM	29
GR/Fe_3O_4NPs	Ciprofloxacin	CPE	Antibiotic plant effluent, tap water and river water	DPV	0.01–20 µM	1.8 nM	59
OCNTs-PDA-Ag	Ciprofloxacin	GCE	$NaNO_3$ and tap water samples	SWV	500 pM - 500 µM	750 Pm	31
Carbon black	Auranofin	GCE	-	LSV	75 nM - 1 mM	150 nM	32
poly(A_2P_2CuC)	Amoxicillin	GCE	human blood serum sample	SWV	2–100 µM	0.0115 µM	60
poly(Alz)	Cefadroxil	GCE	tablet formulation and human urine samples	DPV	0.1–100 µM	0.0081 µM	61
NiO/rGO	Linezolid	Pt	urine samples	DPV	0.001-90 µM	0.0031 µM	62
AgNPs/MnO_2/ErGO	Piroxicam and Ofloxacin	GCE	tap and river waters	DP-AdASV	0.5–90 Mm<br>0.5–170 Mm	0.063 µM<br>0.077 µM	63
NCMO MRs	Ronidazole	GCE	lake and tap water	DPV	0.03–8.82 µM<br>12.83–1221.83 µM	15 nM	64
NiO	Chloramphenicol	CPE	Tap water and Human urine	DPV	100-0.1 µM	1.08×10^{-8} M	65
P/CD@AuNPs	Furazolidone	GCE	human blood serum sample	DPV	0.05–715 µM	2.03 nM	66
ZnO	Hydroxychloroquine	CPE	human urine and tap water	DPV	10^{-3} - 0.8×10^{-6} M	1.33×10^{-7} M	30
$NiFe_2O_4$-NPs	Ceftriaxone	GCE	pharmaceuticals and biological samples	DPV	1.00×10^{-8}–3.20×10^{-6}	1.6 x10^{-9} M	67
Ag/GO	Tetracycline	BDD	-	SWV	-	5 nM	68
GQD/CFX-Ab	Cephalexin	FTO	egg, meat and milk	DPV	-	0.53 fM	69

**Anticancer drugs**							
Flu/dsDNA/MCM41	Flutamide	SPE	blood serum	DPV	0.7 - 10 mM	0.1 mM	70
Nd_2O_5 NPs@GO	Raloxifene	GCE	human serum and urine samples	Chronoamperometry	0.03 - 472.5 µM	18.43 nM	71
MoP-2	Berberine	SPCE	rat plasma	DPV	0.15 - 242.5 uM	0.05 µM	72
dsDNA/poly(CTAB-MWCNTs)	Irinotecan	PGE	peripheral serum	DPV	4–150 µg mL^{-1}	3.06 µg mL^{-1}	73
ds-DNA/PPy/FL Pt/$NiCo_2O_4$	Chlorambucil	PGE	tablet, serum, urine	DPV	0.018–200.0 µM	4 nM	74
SP-Au/MWCNT70@PPy-PTA|dBSA	Methotrexate	SPCE	serum	EIS	0.02–20 µM	1.93 and 7.63 nM	75
Tr/Cp/Fe@rGO/Cu BTC-AIA/CNF	microRNA 155	CPE	human serum	DPV	0.08 fM	0.2 fM–500 pM	76
BSA-Ag/GMT/MIP	Gemcitabine	SPCE	human serum samples	DPV	0.15 µM	0.5 – 200 µmol L^{-1}	77
BiNPs/Gr	Cisplatin	GCE	human serum	DPV	4.4 µM	6.0–180 µM	78
Gr/dsDNA	Irinotecan	SPCE	artificial urine samples	DPV	1.06 nM	1–200 nM	79
CEZLNCs	Etoposide	CPE	human serum and urine sample	DPV	2.7 nM	0.04–120 µM	80
MIP/MWCNTs/Au @Fe_3O_4	Imatinib	CPE	tablet and blood serum	DPV	0.013 µM	1–1000 µM	81
MWCNTs@Fe_3O_4 @-$CuAl_2O_4$	Alpelisib	GCE	human plasma, human urine, tablet	DPV	0.036 µM	0.5–13.6 µM	82
Au NPs/ MWCNTs	Mesna	GCE	plasma and urine	EIS	0.02 nM	0.06 nM - 1.0 nM; 1.0 nM - 130.0 µM	83
ZnO/AMPS@MIP	Sorafenib	GCE	human serum and tablet	DPV	2.25×10^{-13}	1.0×10^{-12} - 1.0×10^{-11}	84
MXene/MOF (ZIF-67)	Flutamide	GCE	human urine and blood serum	DPV	0.015 µM	0.05–70 µM	85
GO	Bevacizumab	GCE	human serum and wastewater fluid	DPV	0.02 µg mL^{-1}	10–1100 µg mL^{-1}	86

Materials: SnO_2/Bi_2S_3: Tin dioxide/bismuth sulfide; AuNPs/RGO: gold nanoparticles/graphene oxide; SDS·Gr: sodium dodecyl sulfate–mediated graphene; r-GOAC: reduced graphene oxide obtained by alternating current; r-GODC: reduced graphene oxide obtained by direct current; $Mn_3O_4/g\text{-}C_3N_4$: graphitic carbon nitride and manganese oxide nanostructures; Gr-PLA: graphite-polylactic acid; $[N(CH_3)_4]_2CoCl_2Br_2$: perovskite compound; KAP/CMWCNTs/CNFs: kanamycin-specific aptamer/ multi-wall carbon nanotubes/ carbon nanofibers; Ni/MoN/MC: composite with cobalt nanoparticles encapsulated in nitrogen-doped carbon nanotube; MIP/Ag/Ni-MOF/N-GQDs: molecularly imprinted polymers / silver nanoparticles/ nitrogen-doped graphene quantum dots; GAgNPs: green silver nanoparticle; PANI/AgBr: functionalized polyaniline-silver bromide; Ag@POM@rGO: silver decorated in polyoxometalate (POM), reduced graphene oxide (rGO) nanocomposite; CNF: carbon nanofibers; Co-TCA@ZIF-67: Co/thiocyanuric acid complex coated ZIF-67 materials; CuMOF: ruthenium doped copper- metal organic framework; GR/Fe_3O_4NPs: graphene nanosheets/ Fe_3O_4 nanoparticles; OCNTs-PDA-Ag: oxygen functionalized carbon nanotubes - electro-depolymerized conductive – polymer; $poly(A_2P_2CuC)$: diaquabis(1,10-phenanthroline)copper(II)chloride complex; poly(Alz): poly(1,2-dihydroxyanthraquinone); NiO/rGO: NiO and reduced graphene oxide nanocomposite; $AgNPs/MnO_2/ErGO$: silver nanoparticles/MnO_2/reduced graphene oxide; NCMO MRs: nickel (Ni^{2+})-doped cobalt molybdate microrods; NiO: nickel oxide; P/CD@AuNPs: polystyrene microspheres decorated with β-cyclodextrin-capped gold nanoparticles; ZnO: zinc oxide; $NiFe_2O_4$-NPs: nickel ferrite nanoparticles; Ag/GO: graphene oxide reduced electrochemically and further electrodecorated with silver; GQD/CFX-Ab: graphene quantum dots/ cephalexin-bovine serum albumin; Flu/dsDNA/MCM41: MCM41 immobilize the short sequence of the p53 tumor suppressor gene; Nd_2O_5 NPs@GO: neodymium sesquioxide nanoparticles decorated graphene oxide; MoP: polypyrrole-molybdenum disulfide nanocomposites; dsDNA/poly(CTAB-MWCNTs): DNA biosensor based on electrodeposited cetyl trimethylammonium bromide-multi-walled carbon nanotubes; ds-DNA/PPy/FL Pt/$NiCo_2O_4$: polypyrrole/flower-like platinum/$NiCo_2O_4$; SP-Au/MWCNT70@PPy-PTA|dBSA: porous multiwalled carbon nano-tube@polypyrrole/polytyramine; Tr/Cp/Fe@rGO/CuMOF/CNF: nanocomposite of carbon nanofiber, metal–organic framework, and magnetic graphene oxide; BSA-Ag/GMT/MIP: silver nanoparticle based probe/gemcitabine/imprinted polymer; BiNPs/Gr: bismuth nanoparticles decorated on a graphene-modified glassy carbon electrode; CEZLNCs: Zno-Lapis lazuli nanocomposite; MIP/MWCNTs/Au@Fe_3O_4: molecularly imprinted polymer/multi-walled carbon nanotubes/Au@Fe_3O_4 nanoparticles; MWCNTs@Fe_3O_4@-$CuAl_2O_4$: multi-walled carbon nanotubes@ iron oxide@ copper aluminum spinel; ZnO/AMPS@MIP: ZnO nanoparticles/2-acrylamido-2-methyl-1-propanesulfonic acid@ molecularly imprinted polymers; MXene/MOF (ZIF-67): $Ti_3C_2T_x$/metal-organic framework nanocomposite.

Electrodes: CC: carbon cloth; BDD: boron-doped diamond electrode; FTO: fluorine-doped tin oxide; GCE: glassy carbon electrode; CPE: carbon paste electrode; SPCE: screen-printed carbon electrode; PGE: pyrolytic graphite electrode; ITO: indium tin oxide electrode.

Techniques: CV: cyclic voltammetry; EIS: electrochemical impedance spectroscopy; DPV: differential pulse voltammetry; SWV: square wave voltammetry; DP-AdASV: differential pulse adsorptive anodic stripping voltammetry; BIA-AD: batch injection analysis with amperometric detection; LSV: linear sweep voltammetry.
